# Knockout of an endogenous mannosyltransferase increases the homogeneity of glycoproteins produced in *Pichia pastoris*

**DOI:** 10.1038/srep03279

**Published:** 2013-11-20

**Authors:** Florian W. Krainer, Christoph Gmeiner, Lukas Neutsch, Markus Windwarder, Robert Pletzenauer, Christoph Herwig, Friedrich Altmann, Anton Glieder, Oliver Spadiut

**Affiliations:** 1 Graz University of Technology, Institute of Molecular Biotechnology, Graz, Austria; 2 Vienna University of Technology, Institute of Chemical Engineering, Research Area Biochemical Engineering, Vienna, Austria; 3 University of Vienna, Department of Pharmaceutical Technology and Biopharmaceutics, Vienna, Austria; 4 Department of Chemistry, University of Natural Resources and Life Sciences, Vienna Austria; 5 Austrian Centre of Industrial Biotechnology (ACIB GmbH), Graz, Austria

## Abstract

The yeast *Pichia pastoris* is a common host for the recombinant production of biopharmaceuticals, capable of performing posttranslational modifications like glycosylation of secreted proteins. However, the activity of the *OCH1* encoded α-1,6-mannosyltransferase triggers hypermannosylation of secreted proteins at great heterogeneity, considerably hampering downstream processing and reproducibility. Horseradish peroxidases are versatile enzymes with applications in diagnostics, bioremediation and cancer treatment. Despite the importance of these enzymes, they are still isolated from plant at low yields with different biochemical properties. Here we show the production of homogeneous glycoprotein species of recombinant horseradish peroxidase by using a *P. pastoris* platform strain in which *OCH1* was deleted. This *och1* knockout strain showed a growth impaired phenotype and considerable rearrangements of cell wall components, but nevertheless secreted more homogeneously glycosylated protein carrying mainly Man8 instead of Man10 N-glycans as a dominant core glycan structure at a volumetric productivity of 70% of the wildtype strain.

The methylotrophic yeast *Pichia pastoris* has long been used for the production of recombinant proteins at high titers. Up to 22 g·L^−1^ have been reported for intracellularly produced recombinant hydroxynitrile lyase^1^ and approximately 15 g·L^−1^ for secreted recombinant gelatin^2^, demonstrating the high production capacity of this microbial host while being able to grow on comparatively simple and inexpensive media. Not only can *P. pastoris* be grown to cell densities as high as 160 g·L^−1^ dry cell weight^3^, it is also capable of performing posttranslational modifications, including the formation of correct disulfide bridges and the glycosylation of secretory proteins, rendering *P. pastoris* specifically suitable for the production of complex eukaryotic proteins^4^.

Glycosylation has long been known to affect various protein properties such as solubility, stability and enzymatic activity (*e.g.*^5^,^6^), which need to be evaluated on a case-by-case basis. Whereas only little is known about O-linked glycosylation, the biosynthesis of N-glycans is well understood. N-glycans are linked to the amido groups of asparagine residues that are recognized by glycotransferases in the sequence motif N-X-S/T, where X is any amino acid but proline. Initially, the biosynthesis steps of N-glycans in yeast and mammals are identical. Dolichol phosphate-linked N-acetylglucosamine (DolP-GlcNAc) is synthesized by the transfer of GlcNAc from uridine diphosphate (UDP) onto DolP on the cytoplasmic side of the ER. After extension to DolP-linked Man_5_GlcNAc_2_, this structure is enzymatically flipped to the ER lumen, where further glucose (Glc) and Man residues are added to form a core glycan, Glc_3_Man_9_GlcNAc_2_, which is transferred to an asparagine within the N-X-S/T sequence motif of a nascent protein chain. Subsequently, the three terminal Glc residues and one Man residue are trimmed by glucosidases I and II and an ER-residing α-1,2-mannosidase to form Man_8_GlcNAc_2_. At this point, the newly formed glycoprotein is transported to the Golgi apparatus, which is where the yeast and mammalian N-glycosylation pathways diverge^7^,^8^,^9^. In the mammalian Golgi apparatus, α-1,2-mannosidases trim the core glycan further to form Man_5_GlcNAc_2_. Ultimately, addition of GlcNAc by a β-N-acetylglucosaminyltransferase I (GnTI), trimming of two further Man residues by a mannosidase II and yet further addition of GlcNAc, galactose (Gal) and sialic acid (Sia) residues by the respective transferases result in the complex N-glycan structures of mammalian proteins^10^. In the yeast Golgi, on the other hand, the Man_8_GlcNAc_2_ glycan is not subjected to further trimming reactions but is substantially extended. In *Saccharomyces cerevisiae*, more than 100 Man residues may account for hypermannosyl N-glycans on secretory proteins. However, the extent of hypermannosylation varies considerably and seems to depend on so far unknown influences, causing vast heterogeneity in the N-glycan pattern of secreted glycoproteins. In *S. cerevisiae* as well as in *P. pastoris* and other yeasts, the first reaction in hypermannosylation is catalyzed by an α-1,6-mannosyltransferase (Och1p) that is encoded by the gene Outer CHain elongation 1 (*OCH1*), which was first discovered and characterized in *S. cerevisiae*^11^,^12^. Och1p uses Man from guanosin diphosphate and links it to the core glycan by an α-1,6-glycosidic bond, forming a substrate that triggers additional mannosylation (Figure 1). Whereas *S. cerevisiae* holds a repertoire of Golgi-resident α-1,2-, α-1,3 and α-1,6-mannosyl and mannosylphosphate transferases, *P. pastoris* seems to lack the Golgi-resident α-1,3-mannosyltransferase, but to possess four additional β-mannosyltransferases instead^7^,^13^,^14^.

Although not as extensive as those of *S. cerevisiae*, the N-glycans of *P. pastoris* are also of the high mannose type and the humanization of the N-glycosylation machinery of *P. pastoris* has been the subject of several studies (Table 1).

Here, we report the deletion of the *OCH1* gene from the *P. pastoris* genome in an irreversible and straight forward approach. Thereby, we generated a new *P. pastoris* platform strain that allows the production of recombinant proteins with shorter glycan structures of considerably increased homogeneity compared to proteins produced in a wildtype strain. In contrast to previous glycoengineering studies, which required several time- and labor-intensive steps of strain engineering, we achieved more homogeneously glycosylated protein with a single gene knockout step. Horseradish peroxidase (HRP) is a versatile enzyme with applications in diagnostics and histochemistry, bioremediation and cancer treatment. However, due to the lack of an appropriate recombinant production process, HRP preparations are still derived from horseradish roots as mixtures of different isoenzymes^15^. In the present study, we produced recombinant HRP in an *och1* knockout strain in the controlled environment of a bioreactor, purified and characterized the enzyme, thus demonstrating the general applicability of this new platform strain by the example of this industrially and medically relevant enzyme.

## Results

### Knockout of *OCH1* from *Pp*ku70- and *Pp*MutS

In yeast, the *OCH1* gene encodes an α-1,6-mannosyltransferase whose activity triggers the subsequent transfer of further mannose and phosphomannose residues onto the N-glycans of secreted proteins in the Golgi apparatus, resulting in heterogeneously hyperglycosylated protein species that appear as a smear on SDS gels, *e.g.*^16^,^17^. This hyperglycosylation not only limits the use of yeast derived proteins as biopharmaceuticals but also greatly impedes traditional downstream processing. Hence, a *P. pastoris* strain that allows the production of less heterogeneously glycosylated proteins would considerably relieve protein production processes with *P. pastoris*.

**Figure 2:**
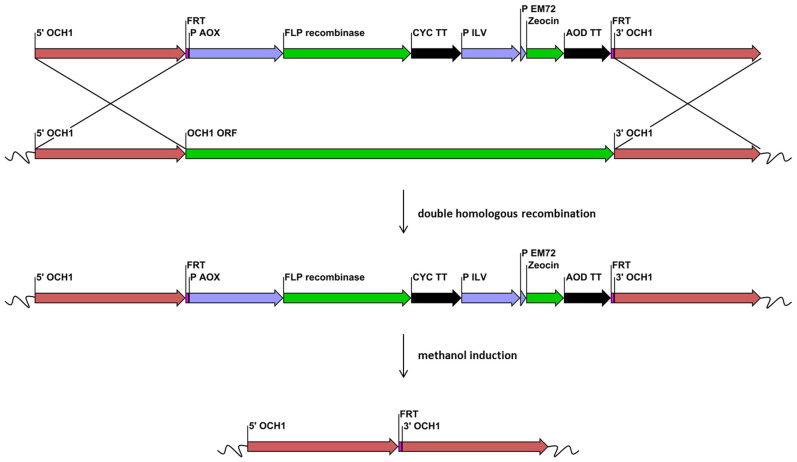
Schematic workflow of the knockout of *OCH1* using a flipper cassette. The regions 5′ OCH1 and 3′ OCH1 represent sequences upstream and downstream of the *OCH1* ORF, respectively. The 34 bp flipper recombinase target (FRT) sequences flank the *AOX1* promoter (P AOX1), the FLP recombinase ORF, the *CYC1* transcription terminator (CYC TT), a constitutive eukaryotic and a prokaryotic promotor (P ILV and P EM72, respectively), a *ble* ORF mediating Zeocin™ resistance and an *AOD* transcription terminator (AOD TT). A double homologous recombination event replaced the *OCH1* ORF in the genome with the flipper cassette. Growth of recombinant cells on methanol induced the production of the FLP recombinase which recognized the two *FRT* sites and excised the inner sequence, leaving only one *FRT* site in the genome. Single fragments are not drawn to scale.

A flipper cassette targeting to the *OCH1* locus was transformed to a *Pp*ku70- strain to replace the *OCH1* open reading frame. This *Pp*ku70- strain has to rely on homologous recombination for gene integration events, in contrast to a wildtype strain^18^. The cassette construct and the knockout workflow are schematically shown in Fig. 2.

Transformation of the flipper cassette to the *Pp*ku70- strain resulted in only few Zeocin™ resistant clones. However, Sanger sequencing of a PCR amplified fragment of the *OCH1* locus from genomic DNA showed that the majority of the tested transformants had correct integration of the transformed cassette. The transformants grew slowly and formed colonies of abnormal shape. This phenotype was preserved when the strains were grown on minimal methanol agar plates to induce the production of the FLP recombinase and subsequent excision of the inner part of the flipper cassette containing the expression cassettes for the FLP recombinase and the Zeocin™ resistance enzyme. Reconstituted sensitivity to Zeocin™, PCR and Sanger sequencing of the former *OCH1* locus confirmed the successful excision of the inner part of the flipper cassette and the efficient replacement of the former *OCH1* ORF with a single *FRT* site of 34 bp.

**Figure 3:**
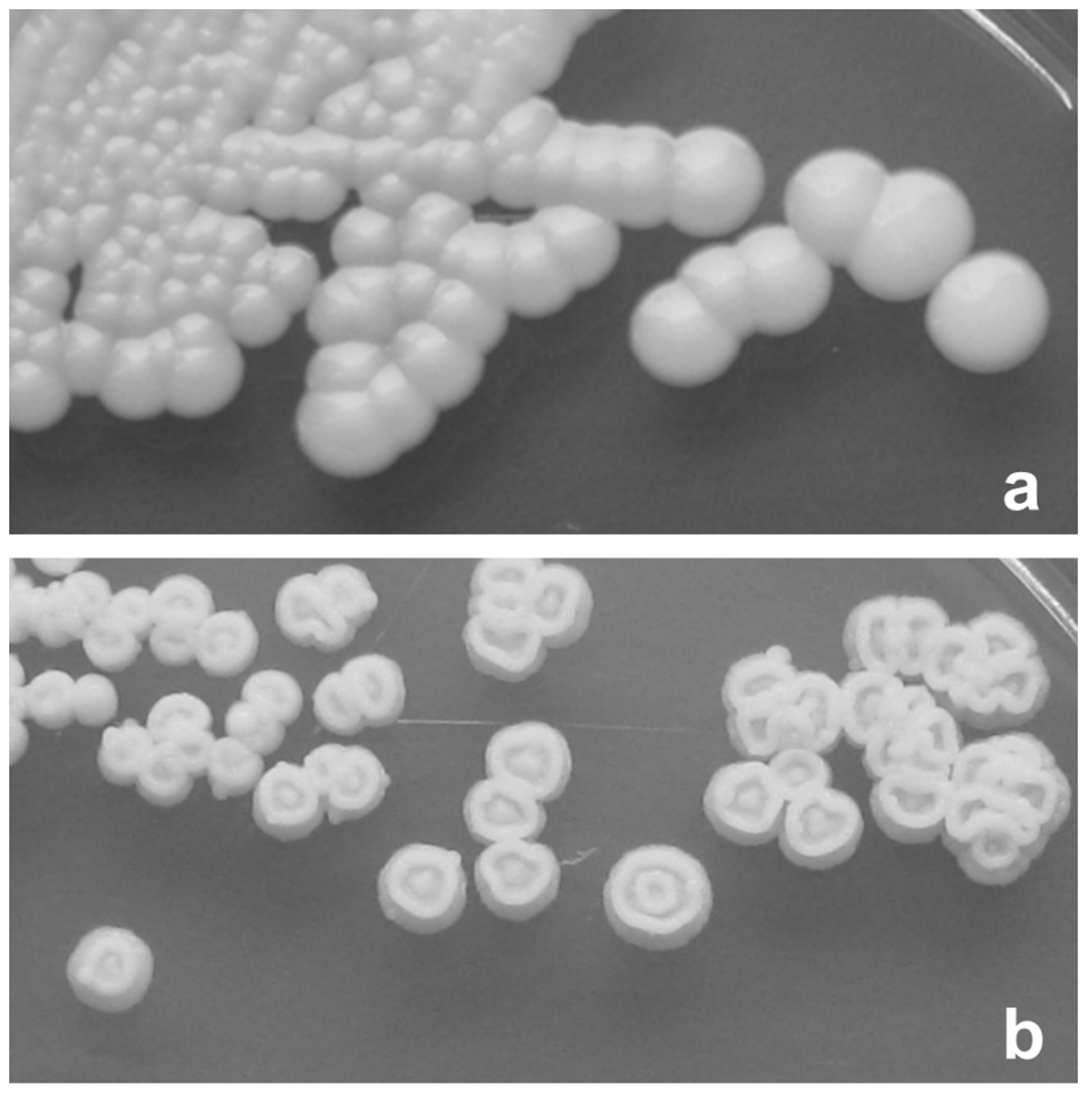
Colony phenotypes. (a), *Pp*MutS; (b), *Pp*FWK3. Both strains were grown on YPD agar.

Transformation of the same *OCH1* flipper cassette to *Pp*MutS resulted in hundreds of clones resistant to Zeocin™. Initial PCR based screenings of over 100 randomly chosen clones did not give any positive hits, analogously to what has been described by Vervecken *et al.*^10^. However, after having identified the corresponding phenotype of positive transformants in the *Pp*ku70- based *och1* knockout strain, designated *Pp*FWK1, also *Pp*MutS based transformants with correct integration of the flipper cassette could be spotted easily on the agar plates since they showed the same unusual colony phenotype as colonies of *Pp*FWK1 (Figure 3).

Increasing the incubation time of the transformed cells on the agar plates to at least four days allowed growth of the *och1* knockout colonies to a size at which their abnormal shape was an obvious hint to their genotype (Figure 3). Again, Zeocin™ sensitivity was reconstituted by induction of the FLP recombinase on minimal methanol agar plates and the replacement of the *OCH1* ORF by a single *FRT* site was shown by PCR and Sanger sequencing. The observed phenotype of the generated *och1* knockout strain *Pp*FWK3 included slow growth, abnormal colony shape and temperature sensitivity at 37°C (data not shown). Upon transformation of the wildtype *OCH1* promoter and *OCH1* ORF to *Pp*FWK3, the detrimental phenotype was found to be complemented. PCR analyses confirmed the unaltered replacement of the former *OCH1* ORF by a *FRT* site, but the presence of the complementing *OCH1* ORF somewhere else in the genome due to ectopic integration of the transformed plasmid pPpT4_BamHI_OCH1rescue.

### Strain morphology

#### Cell morphology and cell division of an och1 knockout strain

**Figure 4:**
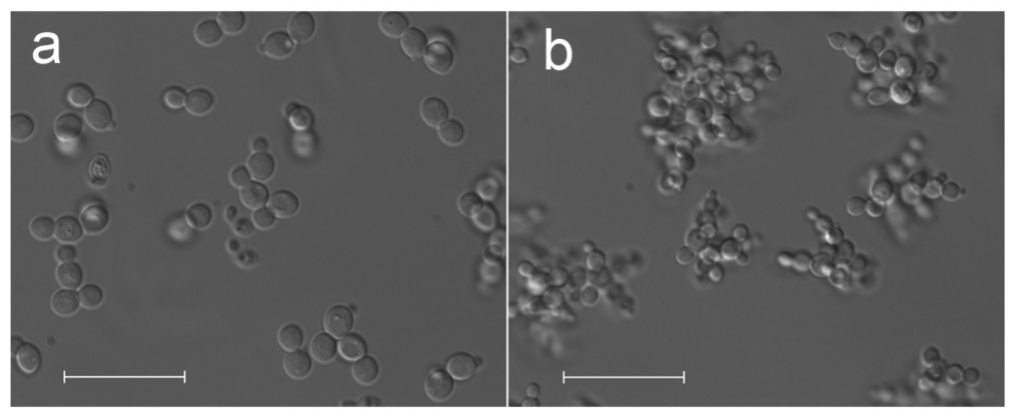
Phenotypic change in *P. pastoris* upon *OCH1* knockout. Representative DIC micrographs of *Pp*Muts and *Pp*FWK3. (a), *Pp*MutS cells in batch culture. (b), covalently linked clusters of multibudded cells in *Pp*FWK3 during the same cultivation phase. Scale bars represent 25 μm.

Consistent with previous reports on Och1p deficient *S. cerevisiae* strains^19^,^20^, we found the N-glycosylation mutant to be characterized by an altered phenotype and growth profile (Table 2). In contrast to the wildtype based strain *Pp*MutS, the *och1* knockout strain *Pp*FWK3 grew in the form of large cell clusters with clumpy appearance and multibudded cells (Figure 4). Daughter cells within these clusters displayed clearly segregated vacuoles, but remained stably attached to the wall of mother cells (Supplementary Figure 1).

#### Knockout induced stress response is reflected by a spatial rearrangement of WGA/STL binding sites

**Figure 5:**
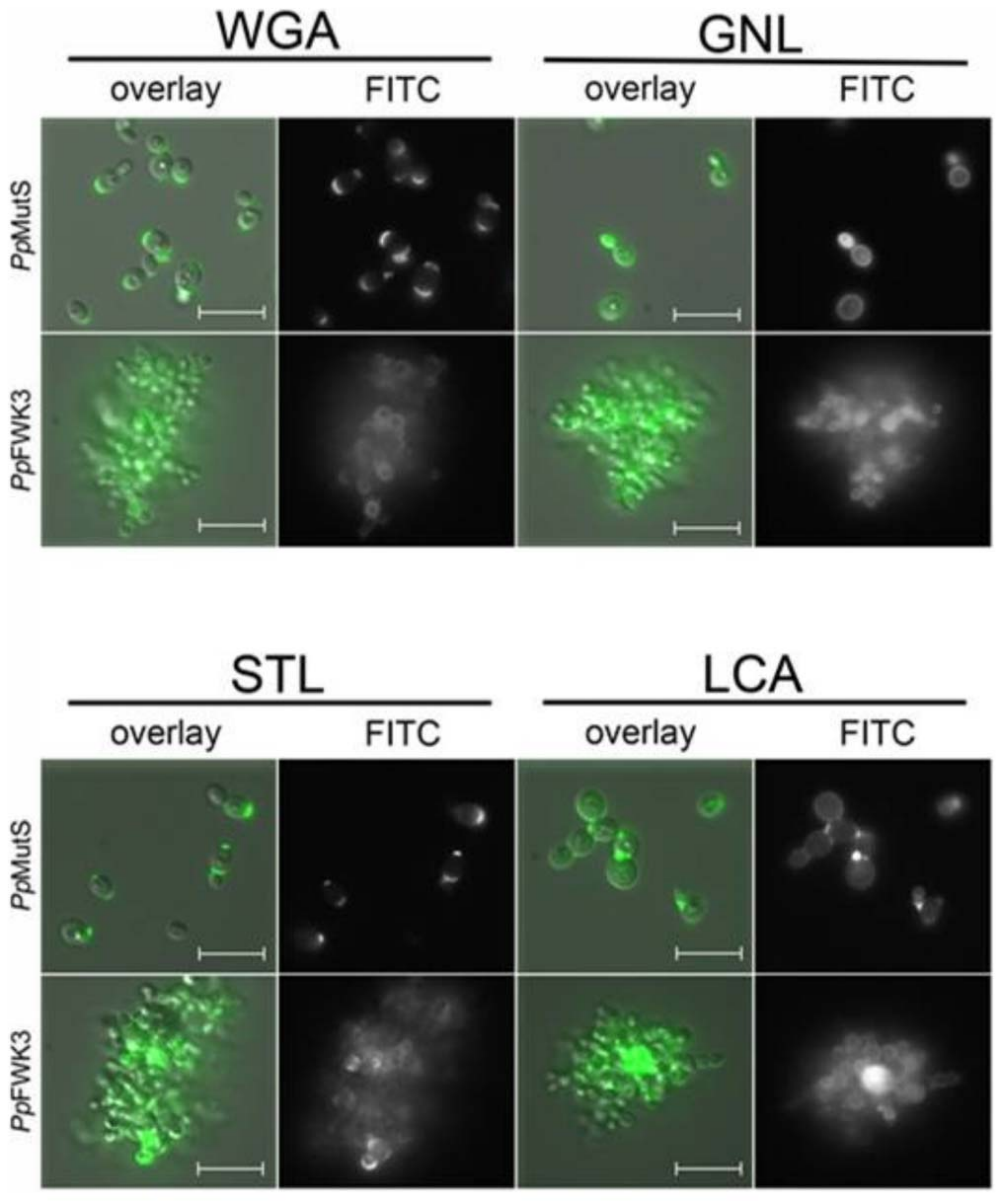
Lectin based glycoprofiling of surface carbohydrate motifs in *Pp*MutS and *Pp*FWK3. Live cells harvested in the exponential growth phase of batch cultivation were incubated with fluorescein labeled lectins. Micrographs show the isolated channel for FITC detection and merged images with DIC (overlay). Scale bars represent 10 μm.

The direct functional implication of the altered N-glycosylation in *Pp*FWK3 for cell morphology and cytokinesis was further illustrated by a striking difference in the chitin deposition after *OCH1* knockout (Supplementary Figure 1). In lectin based glycoprofiling studies, we observed substantially altered binding patterns for the GlcNAc-specific lectin WGA, with the reactive carbohydrate motifs being homogeneously distributed across the entire cell surface in the *och1* knockout strain, instead of remaining confined to the bud scars as in *Pp*MutS (Figure 5 and Supplementary Figure 1).

In order to assess whether a mere alteration in the steric accessibility of chitin chains in the lateral wall was responsible for the difference in the WGA staining behavior or whether chitin was actually specifically localized at bud scars, *Pp*MutS cells were subjected to the same fluorescence microscopic analysis after treatment with concentrated methanol, leading to denaturation of mannoproteins and a substantial increase in cell wall permeability^21^. The efficiency of the cell wall permeabilization protocol was validated by concomitant incubation with a usually non-membrane penetrating DAPI dye, which could readily access the nuclear space after methanol treatment. Still, methanol permeabilized *Pp*MutS cells displayed only the conventional, bud scar selective staining pattern, contrasting to the generalized binding of GNL, which served as a control (Supplementary Figure 2).

**Figure 6:**
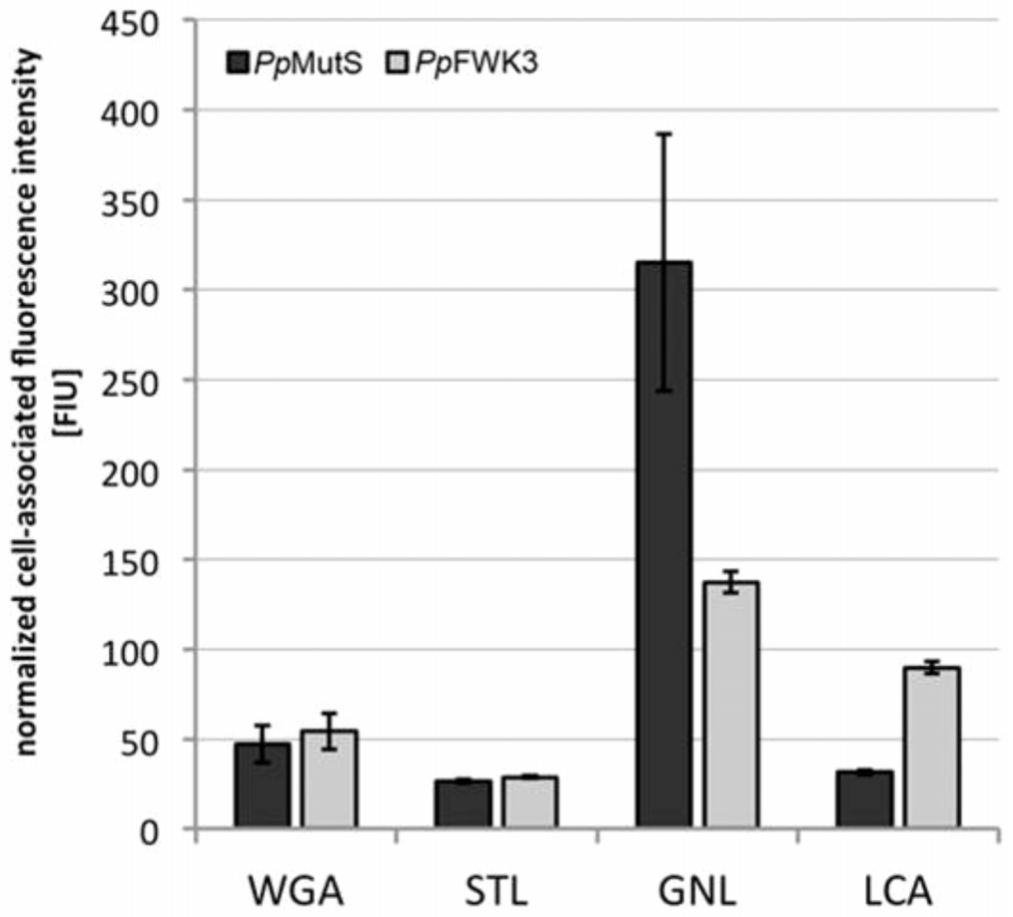
Quantitative determination of lectin binding on *Pp*MutS and *Pp*FWK3 cells. Cell suspensions adjusted to the same concentration level were incubated with FITC labeled lectins. After thorough washing, cells were lysed and the fluorescence intensity of the lysis buffer recorded. Binding data was normalized to the average cellular content of FM® 4–64, which was shown to be similar in both strains via FACS analysis. Values represent mean ± SD of three independent experiments.

Quantitatively, comparative overall ratios of WGA/FM® 4–64 were found for *Pp*MutS and the *och1* knockout strain *Pp*FWK3 (Figure 6), indicating that mainly a spatial redistribution of chitin may be induced by altering the glycosylation machinery, but without general enhancement of the total cellular chitin level. In other words, the rather high chitin concentration at the bud scars in *Pp*MutS seemed to be reduced in favor of an increased chitin deposition in the lateral cell walls of *Pp*FWK3. These results were also confirmed via the chitin binding lectin STL, which has a similar carbohydrate specificity profile as WGA but only limited affinity to isolated GlcNAc residues (Figure 6).

#### Inactivation of the Och1p activity shifted the interaction capacity of mannose specific lectins

The *och1* knockout cells of the *Pp*FWK3 strain displayed a lower binding capacity for the lectin GNL than *Pp*MutS cells (Figure 6). GNL interacts with high mannose N-glycans and preferably reacts with α-1,3-Man residues, but also binds to α-1,6 linked Man residues^22^,^23^. In the current study, the GNL/FM® 4–64 ratio was reduced by 50% in *Pp*FWK3 as compared to *Pp*MutS, but still remained the lectin with highest binding capacity for this strain (Figure 6). In striking contrast, the binding levels of the glycan core-binding lectin LCA were increased upon *OCH1* knockout (Figure 6). Both, GNL and LCA showed an equal distribution across the entire cell wall (Figure 5), corresponding to the established localization of their putative targets, high-mannose N-glycans and core glycans, respectively.

### Production of HRP in the strains *Pp*MutS^HRP^ and *Pp*FWK3^HRP^

To show the applicability of the generated *och1* knockout strain for the production of recombinant proteins, a vector harboring a gene coding for an acidic HRP isoenzyme was transformed into either *Pp*MutS or *Pp*FWK3. Transformation of the linearized constructs into *Pp*FWK3 resulted in fewer Zeocin™ resistant clones than for *Pp*MutS, but sufficient to allow screening for HRP activity after cultivation in a 96-deep well plate in minimal media. Despite the apparent growth defect of *Pp*FWK3, the volumetric yields in HRP activity in these micro-scale cultivations were comparable to those of *Pp*MutS based transformants. Prior to cultivation of *Pp*MutS^HRP^ or *Pp*FWK3^HRP^ in the bioreactor, both strains were analyzed in terms of copy number of the transformed HRP gene to ensure comparability on this level. Both strains were found to have a single copy integration of the HRP encoding gene and were thus considered suitable for comparative bioreactor cultivations.

### Strain characterization in bioreactors

We characterized four *P. pastoris* strains (Table 2) with a recently published method of conducting dynamic experiments during batch cultivations in the controlled environment of a bioreactor^16^,^24^,^25^. After depletion of glucose, a first methanol adaption pulse with a final concentration of 0.5% (v/v) was applied. The adaptation times to the new substrate methanol (Δtime_adapt_), defined as the maximum in offgas activity, were determined for all four *P. pastoris* strains and are shown in Table 2.

**Figure 7:**
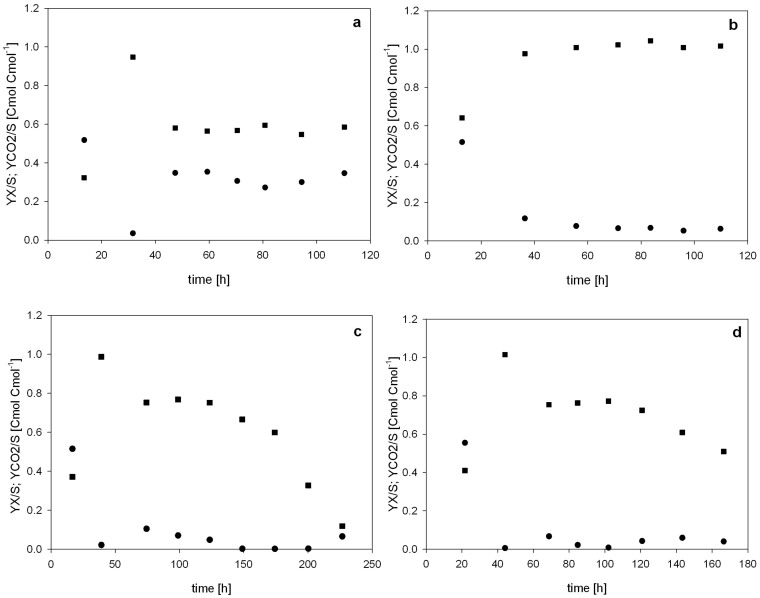
Calculated yields for the different *P. pastoris* strains during batch cultivations with methanol pulses. (a), *Pp*MutS; (b), *Pp*MutS^HRP^; (c), *Pp*FWK3; (d), *Pp*FWK3^HRP^. Black square, carbon dioxide yield (Y_CO2/S_); black dot, biomass yield (Y_X/S_).

The calculated carbon dioxide evolution rate (CER), illustrating the metabolic activity of the different strains, the specific substrate uptake rate (q_s_) and, where appropriate, the specific productivity (q_p_), during the methanol pulses are shown in Supplementary Fig. 3–6. As shown in Supplementary Fig. 3 and 4, the CER profiles for the strains *Pp*MutS and *Pp*MutS^HRP^ showed a similar pattern during the consecutive methanol pulses and q_s_ values stayed constant over time. In contrast, the CER profiles for the strains *Pp*FWK3 and *Pp*FWK3^HRP^ substantially changed over time (Supplementary Figures 5 and 6). After each methanol pulse, less CO_2_ was produced per time and volume, indicating that the *P. pastoris* cells became metabolically less active. Thus, the consumption of 1% (v/v) methanol took longer after each consecutive pulse (compare Supplementary Figures 3 and 4 with Supplementary Figures 5 and 6). The altered metabolic activity of the *och1* knockout strains was also depicted in the calculated yields (Y_X/S_ and Y_CO2/S_), which are shown in Fig. 7.

For the strains *Pp*MutS and *Pp*MutS^HRP^ both the carbon dioxide yield (Y_CO2/S_) and the biomass yield (Y_X/S_) stayed constant during the six conducted consecutive methanol pulses (Figure 7a and b). Evidently, the insertion of the HRP gene into strain *Pp*MutS affected its physiology as Y_X/S_ decreased, whereas Y_CO2/S_ increased (compare Figure 7a and b). Hence, *Pp*MutS^HRP^ mainly used the substrate methanol for protein production and dissimilation than for biomass growth.

Interestingly, the calculated yields for *Pp*FWK3 and *Pp*FWK3^HRP^ strains showed a very different behaviour. Although Y_X/S_ was again rather constant, Y_CO2/S_ decreased dramatically in the course of the six to seven consecutive methanol pulses (Figure 7c and d), indicating that these two strains became more and more metabolically inactive. Since HPLC analysis revealed that no undesired metabolites were produced in substantial amounts during any of the four cultivations, the C-balances for strains *Pp*FWK3 and *Pp*FWK3^HRP^ were determined close to 1.0 only at the beginning of the cultivation but rapidly decreased over time, whereas the C-balances for strains *Pp*MutS and *Pp*MutS^HRP^ were always determined to be close to 1.0 (Supplementary Figure 7). A summary of the determined strain specific parameters of the four different *P. pastoris* strains is given in Table 2.

As shown in Table 2, the maximum specific growth rates on glycerol (max. μ_Gly_) for *Pp*FWK3 strains were approximately 1.5-fold lower than for *Pp*MutS strains. The yields on glycerol showed a similar pattern for *Pp*MutS and *Pp*FWK3 strains, as the yields were shifted towards production of carbon dioxide rather than biomass when the strains were hosting the gene for recombinant HRP. Both *Pp*FWK3 strains needed less than half of the adaptation time to methanol (Δtime_adapt_) compared to the *Pp*MutS strains. The altered glycosylation machinery in the *Pp*FWK3 strains seems to allow a faster adaption to methanol, which could be of great significance for industrial applications where fast and efficient bioprocesses are required. As expected, the adaptation times of the strains hosting the recombinant enzyme were longer compared to the strains not carrying this additional gene. The average specific substrate uptake rates for both substrates (q_s_) glycerol and methanol were lower for the *Pp*FWK3 strains than for *Pp*MutS strains. In respect to the maximum specific uptake rate for methanol (max. q_MeOH_) the values for the different strains were quite similar. However, these values were determined during the 3^rd^ and the 5^th^ methanol pulse for *Pp*MutS and *Pp*MutS^HRP^, respectively. The values during the other pulses were alike. For *Pp*FWK3 strains on the other hand, these maxima could only be determined during the 1^st^ methanol pulse. After that, the values for q_s_ constantly decreased, indicating a progressional reduction of the metabolic activity of the *Pp*FWK3 strains during consecutive methanol pulses. This was also underlined by constantly decreasing Y_CO2/S_ and C-balances. However, despite this seemingly negative impact, *Pp*FWK3^HRP^ produced the HRP isoenzyme at specific and volumetric productivities which were only reduced by 35% and 30%, respectively, compared to *Pp*MutS^HRP^. In a previous study, we introduced the efficiency factor η, which puts the productivity of the strains in direct relation to the consumed substrate^26^. In this respect, the *och1* knockout strain *Pp*FWK3^HRP^ even showed a higher ratio than *Pp*MutS^HRP^ and thus proved to be of justifiable interest for the production of recombinant proteins.

### Enzyme purification and characterization

**Figure 8:**
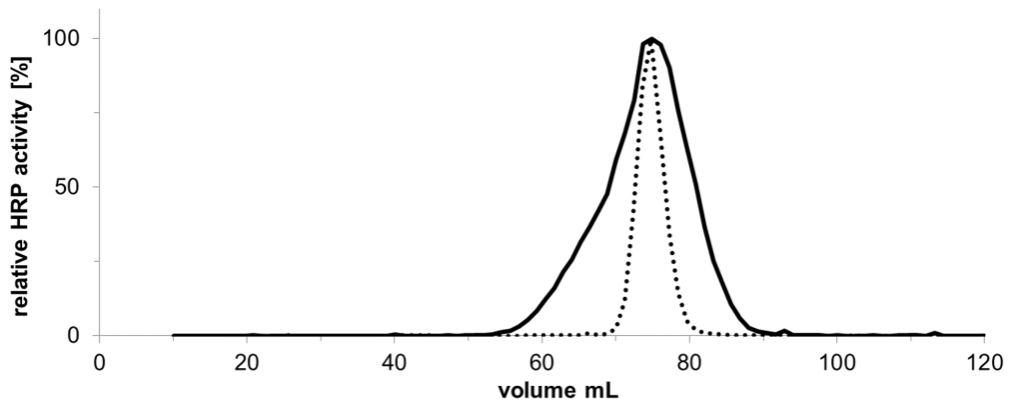
Size exclusion chromatogram of HRP glycovariants. Solid line, HRP produced in *Pp*MutS^HRP^; dashed line, HRP produced in *Pp*FWK3^HRP^. The run was performed at a flow of 9 cm·h^−1^ and fractions of 1.2 mL were collected. The measured HRP activities per fraction are shown as relative activities with the respective maximum activities set to 100% for better comparability. The unnormalized maximum activities were 2.6 and 15.1 U·mL^−1^ for HRP from *Pp*MutS^HRP^ and from *Pp*FWK3^HRP^, respectively. The loaded volume was approximately 200 μL for HRP from either *Pp*MutS^HRP^ or *Pp*FWK3^HRP^.

In order to demonstrate the successful decrease of heterogeneity in the glycosylation of HRP when produced in an *och1* knockout strain, size exclusion chromatography (SEC) was performed with cell free cultivation broth (Figure 8). A SEC elution profile has higher specificity and sensitivity than an image of an SDS polyacrylamide gel and was therefore preferably used for this purpose. HRP produced in *Pp*MutS^HRP^ eluted with higher heterogeneity than HRP produced in *Pp*FWK3^HRP^.

**Figure 9:**
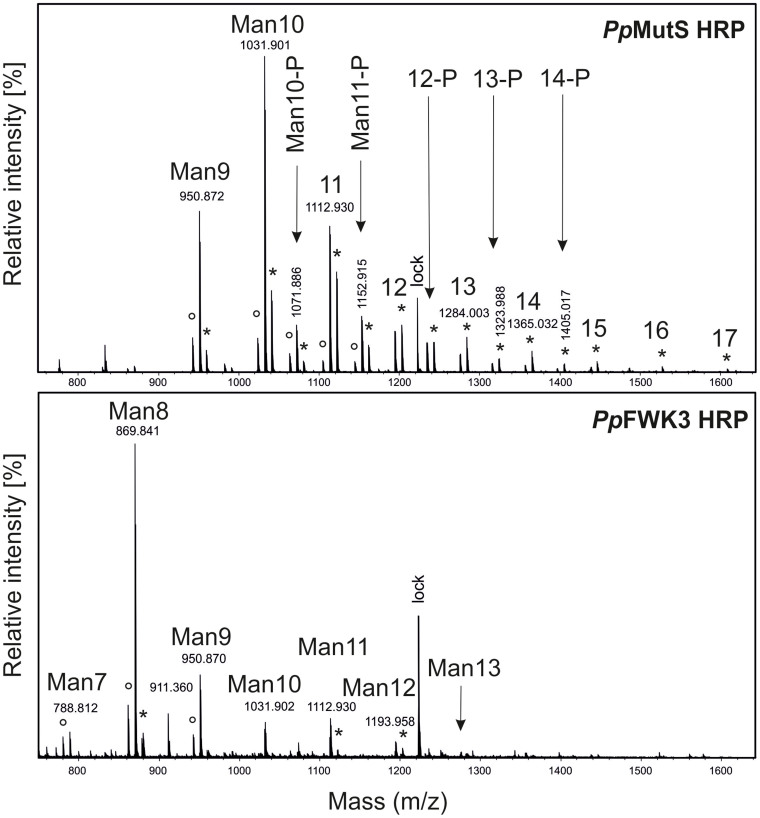
Chromatogram of liquid chromatography-mass spectrometry of glycans released from HRP produced in either *Pp*MutS^HRP^ or in *Pp*FWK3^HRP^. HRP [M + 2H]^2+^ and [M + 2NH_4_]^2+^ ions are marked with ° and *, respectively. “Man10-P” or just “12-P” indicate phosphorylated glycans. lock, lock mass.

Judging by the size exclusion chromatogram (Figure 8), HRP produced in a *Pp*MutS^HRP^ strain substantially differed in its surface glycosylation pattern compared to HRP produced in a *Pp*FWK3^HRP^ strain, which showed higher homogeneity. To analyze this phenomenon in detail, we enzymatically released the glycans from the produced recombinant HRPs and analyzed them via liquid chromatography-mass spectrometry (LC-MS). Reducing glycans were observed mainly as doubly charged [M + H + NH_4_]^2+^ ions (Figure 9). Analysis of enzymatically released glycans from HRP produced in either *Pp*MutS^HRP^ or *Pp*FWK3^HRP^ confirmed the expected decrease in both N-glycan size and heterogeneity. The dominant core glycan structure shifted from Man10 to Man8 in strain *Pp*FWK3^HRP^ (Table 3). As shown in Fig. 9, HRP produced in the strain *Pp*MutS^HRP^ carried a greater variety of different glycan chains consisting of up to 17 mannoses and a higher amount of phosphorylated sugars than HRP produced in *Pp*FWK3^HRP^.

The evaluation of the relative peak areas underlined this observation (Table 3), as around 60% of the identified glycan structures cleaved off from HRP produced in the *och*1 knockout strain *Pp*FWK3^HRP^ were of the Man8 type, whereas no structure of that type was identified for HRP from *Pp*MutS^HRP^. As shown in Table 3 there were also much more different glycanchains identified on HRP from *Pp*MutS^HRP^ and interestingly no phosphorylated mannose structures were found on HRP from *Pp*FWK3^HRP^.

To check whether the kinetic constants or the stability of the enzyme were affected by the altered glycosylation pattern, we characterized purified preparations of HRP. HRP preparations produced by either *Pp*MutS^HRP^ or *Pp*FWK3^HRP^ in bioreactor cultivations were purified by using a recently described strategy for HRP isoenzyme C1A^27^. Both HRP preparations did not bind to the mixed mode HCIC resin but were found in the flowthrough, *i.e.* 93% of HRP produced in *Pp*MutS^HRP^ and 87% of HRP produced in *Pp*FWK3^HRP^. Contaminating proteins were retained on the resin, leading to a partial purification at a factor of approximately 2.5 for both enzyme solutions. A subsequent size exclusion step gave an additional purification factor of approximately 2.0. After purification, the fractions with the highest purification factor were pooled and ultrafiltrated. The enzyme HRP produced in *Pp*MutS^HRP^ was concentrated to around 3.0 mg·mL^−1^, whereas HRP produced in *Pp*FWK3^HRP^ could not be concentrated due to the immediate formation of precipitates during ultrafiltration and the resulting clogging of the membrane, indicating a reduced solubility of the extracellular proteins in this preparation. We determined the kinetic constants for both enzyme preparations with H_2_O_2_ as electron donor at saturating concentration and ABTS as electron acceptor in varying concentrations (Table 4; Supplementary Figure 8).

As shown in Table 4, the affinity of HRP towards the substrate ABTS was increased, as the K_M_ was found to be decreased by approximately 15% by the altered surface glycosylation. However, V_max_ was decreased by nearly 20%. Also, the thermal stability of the produced HRP glycovariants at 60°C was studied (Supplementary Figure 9) and the half life times (τ_1/2_) were determined^28^. The τ_1/2_ of HRP produced in *Pp*MutS^HRP^ was determined with 384 s, whereas HRP from *Pp*FWK3^HRP^ showed a reduction in its thermal half life time of around 50% with 198 s.

## Discussion

Despite the numerous advantages of using *P. pastoris* as a host organism for recombinant protein production, its inherent heterogeneous yeast type hyperglycosylation of secreted proteins has to be addressed by extensive and elaborated strain modifications. Here, we present a straight forward approach for the generation of a wildtype based *P. pastoris* platform strain that allows the production of more homogeneously glycosylated recombinant proteins due to an irreversible deletion of the *OCH1* gene.

Yeast hypermannosylation largely depends on the initial activity of an α-1,6-mannosyltransferase in the Golgi apparatus. Elimination of this activity was achieved by replacement of the *OCH1* ORF with a single 34 bp *FRT* site by using a flipper cassette. However, this approach required double homologous recombination at the correct locus in the genome. Unfortunately, homologous integration events only play a minor part in *P. pastoris*, as recently demonstrated by Näätsaari *et al.*^18^ and which was found to be especially true for the *OCH1* locus by Vervecken *et al.*^10^. A new *P. pastoris* strain with inactivated non-homologous end joining pathway, designated *Pp*ku70-, proved to be a particularly convenient tool to identify the phenotype of the specific knockout strain in this study. Since homologous integration was the sole possibility for recombination events in the *Pp*ku70- strain, the total number of positive transformants was predominantly made up by transformants with homologous integration. The fact that particularly few colonies were obtained by targeting of the transformed flipper cassette to the *OCH1* locus when using the *Pp*ku70- strain also supported the hypothesis of increased difficulty of homologous recombination in that locus.

An *och1* knockout strain of *S. cerevisiae* was described to show several defects such as impaired budding and increased temperature sensitivity^11^. Choi *et al.* mentioned temperature sensitivity and increased flocculation for their *P. pastoris* *och1* knockout strain^13^, but neither they nor Vervecken *et al.*^10^ described any further severe growth defects. However, the *och1* knockout strain in the present study was found to show not only formation of cell clusters and temperature sensitivity, but also decreased growth which might be due to an impaired cell wall structure and thus complicated bud formation. Similarly, a recently generated *och1* knockout strain based on the *his4* mutant strain *P. pastoris* GS115 was described with slower growth and rough colony surface^29^. Explanations for these divergent findings remain speculative, but might be due to single nucleotide polymorphisms and hence different strain backgrounds. Also, a secondary integration event of the transformed flipper cassette cannot be completely excluded. However, considering that as little as 100 ng of the cassette were transformed and that all clones that exposed the described phenotype had the correct integration of the cassette in the *OCH1* locus indicates that the observed phenotype can be ascribed rather to the knockout of *OCH1* than to any additional genomic rearrangement. Also, the similarity of the observed phenotype in the present *P. pastoris* *och1* knockout strain to the phenotype described for a *S. cerevisiae* *och1* knockout strain^11^ very much suggests that the deletion of the *OCH1* gene is actually responsible for the phenotype observed in this study. Most strikingly, reintroduction of the wildtype *OCH1* gene to the *och1* knockout strain *Pp*FWK3 restored its phenotype, thus conclusively linking the observed phenotype of *Pp*FWK3 to the deletion of the *OCH1* gene. Transformation of the *och1* knockout strain with a linearized vector harboring an expression cassette for the production of an HRP isoenzyme via electroporation was found to result in lower transformation efficiency than electroporation of a *P. pastoris* wildtype strain. This increased sensitivity to electroporation might be another reflection of the altered cell wall composition that could be shown by lectin based glycoprofiling.

The highly specific interaction of the lectin WGA with the exposed chitin ring of bud scars^30^ has previously been reported for other yeasts and can be used for the precise determination of the number of cell divisions performed^31^,^32^. To the best of our knowledge, this is the first confirmation of this structure-related specificity for *P. pastoris*. However, in the *och1* knockout strain, we noticed an almost complete loss of bud scar selectivity. The reasons for the regionally diverse distribution of chitin in *Pp*FWK3 may either lie in an increased accessibility of previously cryptic chitin chains in the lateral cell wall (*i.e.* when the protective polymannan layer is missing), or in an actively increased chitin synthesis and deposition at the cell wall, *i.e.* as a compensatory response to the cell wall stress caused by the impaired barrier function. Such stimulation of counter-regulatory pathways upon impairment of cell wall integrity has been observed in several yeast species^33^,^34^ and involves diverse mechanisms and signaling cascades^35^, which are believed to be directly or indirectly connected to the deletion of Och1p activity^20^. Especially the osmotic stress exerted on the cell as a result of the impaired cell wall integrity is known to present an important factor for the induction of counter-regulatory pathways^36^. Due to the persistence of bud scar specific binding of WGA in methanol treated *Pp*MutS cells, we concluded that the strong affinity of WGA to the overall cell wall of *Pp*FWK3 cells traced back to a *de novo* deposition of carbohydrate epitopes (most probably chitin) in this *och1* knockout strain, and not an increased exposure of constitutive cell wall glycans that are invariably present but usually shielded by an outer chain hypermannan structure in the wildtype strain. The somewhat reduced STL/FM® 4–64 ratio compared to the WGA/FM® 4–64 ratio may be connected to the minor differences regarding the preferentially binding ligand. Based on the similarity between STL and WGA staining patterns, it is unlikely that any GlcNAc motifs other than chitin (*e.g.* in the core glycan of mannoproteins) were the primary binding epitopes detected by either of the two lectins. As a side aspect of the current work, we were able to demonstrate the use of fluorescence labeled lectins as convenient and versatile probes for visualizing stress responses derived from impaired cell wall integrity in yeast. The decreased GNL/FM® 4–64 ratio of *Pp*FWK3 compared to *Pp*MutS could be explained by the minimized amount of high mannose N-glycans and the therefore inherently lower amount of potential GNL target ligands (*i.e.* α-1,3- and α-1,6-Man residues) of *Pp*FWK3 compared to *Pp*MutS. Nevertheless, the remaining core glycan provided sufficient GNL targets for a distinct signal. The increased LCA/FM® 4–64 signal of *Pp*FWK3 on the other hand, may be explained by the preference of this lectin for short chain X-α-1,2-Man–Man motifs, with X representing either α-Man or β-GlcNAc^37^. In mannoproteins, such short motifs may be found in the core glycans, the accessibility of which may be enhanced in absence of the usually highly branched polymannan structures of a *P. pastoris* wildtype strain^33^,^34^.

The detailed characterization of the different *P. pastoris* strains in the controlled environment of a bioreactor revealed that the *och1* knockout strains were physiologically impaired compared to their wildtype equivalents. During the consecutive pulses, the carbon dioxide yield Y_CO2/S_ and the C-balances constantly decreased, indicating a loss in metabolic activity. This was also apparent in the CER signals during the single methanol pulses (Supplementary Figure 5 and 6). At the beginning of the cultivation, methanol was metabolized much faster than during later methanol pulses. We followed the morphology of the *P. pastoris* cells during cultivations via microscopy and identified formation of cell clusters by the *och1* knockout strains. Obviously, the altered surface glycosylation of *och1* knockout cells also affected the budding process. Instead of budding off, the daughter cells stayed attached to the mother cell. The microscopically observed increased tendency for cluster formation may be regarded as an effect which is intrinsically linked to the loss of the polymannan layer upon Och1p inactivation. Cell disruption experiments showed that neither treatment with Triton X-100, 0.5% EDTA or 5 M urea, nor extensive mechanic shearing via sonication allowed breaking the clusters to single cells. Thus, we conclude that deficiencies in the constitutive cell division machinery upon Och1p inactivation led to a strong, covalent linkage between the cell walls, which remained intact even after the end of the normal budding process. The exact nature of this linkage remains speculative at the current point, but may be associated to aberrant glycosylation steps in the glycan backbone, occurring as a compensatory adaptation to the lack of Och1p activity^20^,^38^. Due to these very dense and compact formations we hypothesize that the cells in the center of these clusters became limited in oxygen and nutrients and showed no more metabolic activity. Since these cell clusters increased in size over time, the overall metabolic activity of the total amount of cells in the bioreactor decreased, which we observed in decreasing Y_CO2/S_ and C-balances. However, these cell clusters were still able to produce recombinant protein. In fact, both the specific productivity and the volumetric productivity, the main focus of industrial bioprocesses, were only reduced by 35% and 30%, respectively, compared to the *P. pastoris* strain with an intact *OCH1* gene. However, due to the constant decrease in metabolic activity over time, use of the *och1* knockout strain in industrial processes will require considerable modifications to current standard protocols to optimize recombinant protein production in this strain.

When we analyzed the HRP produced by either a *Pp*MutS^HRP^ strain or the *och1* knockout strain *Pp*FWK3^HRP^, we found the enzyme preparation from *Pp*FWK3^HRP^ to be considerably more homogenously glycosylated. More detailed analyses of the surface glycan chains of HRP by mass spectrometry revealed striking differences in the glycosylation pattern between HRP produced in *Pp*MutS^HRP^ and *Pp*FWK3^HRP^. HRP produced in *Pp*MutS^HRP^ carried a more heterogeneous glycopattern with several high-mannose structures and a great amount of phosphorylated sugars. The most dominant glycan was found to be a Man10 structure. In contrast, the most dominant glycan of HRP produced in *Pp*FWK3^HRP^ was a Man8 core glycan structure. This reduction is in agreement with our expectations since no Och1p could act on Man_8_GlcNAc_2_ core glycan structures in the Golgi of the *Pp*FWK3^HRP^ strain as it could in *Pp*MutS^HRP^. Due to the missing Och1p activity, other glycosyltransferases, especially mannosylphosphate transferases, were reduced in their activity resulting in a more homogeneous glycopattern on the surface of recombinantly produced HRP from *Pp*FWK3^HRP^ (Table 3).

Enzymatic characterizations of HRP revealed an increase in the affinity to the substrate ABTS, but a decrease of V_max_ for HRP with a more homogeneous glycopattern. Also, a decreased stability at 60°C for the homogeneously glycosylated HRP compared to the heterogeneously hyperglycosylated glycovariant was observed.

In conclusion, we irreversibly eliminated the *OCH1* encoded α-1,6-mannosyltransferase activity of *P. pastoris*. The phenotype of the generated *och1* knockout platform strain resembled the phenotype described for the same knockout in *S. cerevisiae*. Nevertheless, the strain was successfully employed for the production of recombinant HRP as a reporter enzyme. Strain specific parameters were determined in comparative bioreactor cultivations. Recombinant HRP from either an unaltered *P. pastoris* strain or the *och1* knockout strain was purified and characterized. The main findings of this study can be summarized as:

- The *och1* knockout strains were characterized by slow growth, increased temperature sensitivity and formation of cell clusters. The altered N-glycosylation pathway and resultant structural impacts in the *Pp*FWK3 strain appears to have triggered the dynamic reorganization of surface mannose residues and other glycan structures. The cellular response seemed to be more diverse than just a simple lack of an outer chain hypermannan structure, and may also involve secondary counter-regulatory mechanisms on the metabolic or structural level^20^,^38^. Our results in this regard are in direct agreement with previous work on *och1* deletion strains of *S. cerevisiae*^19^,^39^. Lectin based glycoprofiling represented a rapid and reliable method to provide functional proof for the successful deletion of Och1p activity in *P. pastoris*.
- In the course of consecutive methanol pulses, the *och1* knockout strains lost their metabolic activity due to the formation of cell clusters, thus making the adaption of current production processes necessary.
- As shown by detailed LC-MS data, the produced recombinant enzyme exhibited a more homogeneous surface glycosylation, which is beneficial for subsequent downstream processing and applications.
- V_max_ with ABTS as substrate and the thermal stability at 60°C were reduced for the homogeneously glycosylated HRP, whereas its affinity for ABTS was increased, rendering the enzyme suitable for most applications.

Here, we report the thorough biotechnological characterization of a *P. pastoris* platform strain that allows the production of recombinant proteins with considerably increased homogeneity in their glycosylation pattern due to an irreversible knockout of the *OCH1* gene. Currently, efforts are driven forward to elucidate the potential benefits of the cell morphological changes in glycoengineered *P. pastoris* strains for the expression of recombinant proteins^40^. Also, future studies will focus on rescuing the growth impaired phenotype to generate a strain that shares the favorable growth phenotype of a wildtype strain but still allows the production of homogeneously glycosylated secreted proteins.

## Methods

### Chemicals

Enzymes and deoxynucleotide triphosphates were obtained from Thermo Scientific (formerly Fermentas, Germany). Phusion™ High-Fidelity DNA-polymerase was from Finnzymes (Finland). 2,2′-azino-bis(3-ethylbenzthiazoline-6-sulfonic acid) diammonium salt (ABTS) was purchased from Sigma-Aldrich (Austria). Difco™ yeast nitrogen base w/o amino acids (YNB), Difco™ yeast nitrogen base w/o amino acids and ammonia sulfate (YNB2), Bacto™ tryptone and Bacto™ yeast extract were purchased from Becton Dickinson (Austria). Zeocin™ was purchased from InvivoGen (France) via Eubio (Austria). Fluorescein isothiocyanate (FITC) labeled lectins for microscopic analysis were obtained from Vector Laboratories (USA), comprising wheat germ agglutinin (WGA) from *Triticum vulgaris*, *Lens culinaris* agglutinin (LCA), *Galanthus nivalis* lectin (GNL) and *Solanum tuberosum* lectin (STL). FM® 4–64 membrane stain and Hoechst 33342 or 4′,6-diamidino-2-phenylindole (DAPI) nucleic acid stains were purchased from Life Technologies (USA). Other chemicals were obtained from Carl Roth (Germany).

### Microorganisms

DNA manipulations were performed in accordance to standard protocols^41^ in *E. coli* Top10F' (Life Technologies, formerly Invitrogen, Austria). All *P. pastoris* strains in this study were based on the wildtype strain CBS 7435 (identical to NRRL Y-11430 or ATCC 76273). Initial *OCH1* knockout studies were performed in a *ku70* deletion strain, previously described by Näätsaari *et al.*^18^, hereafter called *Pp*ku70-. Since *P. pastoris* strains with Mut^S^ phenotype have been repeatedly shown to be superior over strains with Mut^+^ phenotype for the production of recombinant proteins (*e.g.*^26^), the ultimate *och1* knockout strain was based on a Mut^S^ strain described in^18^, hereafter called *Pp*MutS.

### Deletion of the *OCH1* gene

Based on the genome sequence of the *P. pastoris* wildtype strain CBS 7435^42^, the primers OCH1-5int-fw1 and OCH1-5int-rv1 were designed to amplify a DNA fragment upstream the *OCH1* open reading frame from genomic DNA isolated according to^43^. Primers OCH1-3int-fw1b and OCH1-3int-rv1b were designed to amplify a fragment downstream of the *OCH1* ORF. OCH1-5int-rv1 and OCH1-3int-fw1b were designed to add sequences that overlap with the *FRT* flanked inner part of a flipper cassette. All primer sequences are listed in Table 5.

The two *OCH1* targeting fragments of approximately 1.5 kb each were used to assemble a flipper cassette via overlap extension PCR^18^. Transformation of 100 ng of the assembled flipper cassette into either *Pp*ku70- or *Pp*MutS was performed as described by Lin-Cereghino *et al.*^44^. Transformants were identified on yeast extract-peptone-dextrose (YPD) agar plates containing 100 mg·L^−1^ Zeocin™. Double homologous recombination of the flipper cassette in the *OCH1* locus was verified by PCR using the primers OCH1check-fw1 and OCH1check-rv2 (Table 5) and Sanger sequencing, using isolated genomic DNA as template. Expression of the FLP recombinase gene was induced by growing positive transformants on minimal methanol agar plates. The FLP recombinase mediated excision of the *FRT* flanked inner part of the flipper cassette was shown by restored sensitivity of the cells towards Zeocin™ and again by PCR and Sanger sequencing. The resulting *Pp*ku70- *och1* knockout strain was designated *Pp*FWK1, the *Pp*MutS *och1* knockout strain was designated *Pp*FWK3.

Complementation of the observed phenotype of the *Pp*FWK3 strain was performed by transforming a plasmid that was constructed by assembly^45^ of two fragments, which were generated by PCR using the primers OCH1rescue-fw1 and OCH1rescue-rv1 using genomic DNA from *Pp*MutS as template, and OCH1rescue_T4fw and OCH1rescue_T4rv using the plasmid pPpT4_S^18^ as template (Table 5). The resulting plasmid contained the wildtype *OCH1* ORF plus 698 bp of upstream sequence, putatively harboring the natural *OCH1* promoter, and was designated pPpT4_BamHI_OCH1rescue. Approximately 500 ng of *BamHI* linearized plasmid were transformed to *Pp*FWK3, aliquots were plated on YPD Zeocin™ agar plates and incubated at 28°C for two days. PCR with the primers OCH1check-fw1 and OCH1check-rv2 from isolated genomic DNA of transformant strains was performed to confirm the unaltered replacement of the former *OCH1* ORF by a single *FRT* site. A second PCR with the primers OCH1-ORF-fw and OCH1-ORF-rv from the same genomic DNA was performed to confirm the presence of a plasmid transmitted *OCH1* ORF somewhere else in the genome. A resulting strain with restored wildtype phenotype was designated *Pp*FWK3^R^.

### Phenotypic strain characterization

#### Lectin based glycoprofiling via fluorescence microscopy

Qualitative analysis of lectin binding was performed by incubating 500 μL of *Pp*MutS or *Pp*FWK3 cell suspensions in 20 mM HEPES buffer, pH 7.4, at an OD_600_ of 0.3 with 500 μL of the respective lectin solution (250 pmol·mL^−1^ in 20 mM HEPES, pH 7.4) for 30 min at 4°C. If appropriate, 5 μg·mL^−1^ HOECHST 33342 nucleic acid stain or 0.5 μg·mL^−1^ FM® 4–64 membrane stain were included in the incubation mix. After thorough washing by repeated centrifugation (1700 × g, 5 min) and resuspension, cells were diluted in 1.0 mL of particle free phosphate buffered saline (PBS; 50 mM, pH 7.4) and mounted in FlexiPERM® coverslip 12-well plates for microscopic analysis. Images were acquired on a Zeiss Epifluorescence Axio Observer.Z1 deconvolution microscopy system (Carl Zeiss, Germany) equipped with LD Plan-Neofluar objectives and the LED illumination system Colibri®. Exposure wavelengths and filter sets of the individual channels were chosen according to the respective fluorophore(s) (DAPI/Hoechst 33342: ex/em 365/450 nm; FITC: ex/em 485/525 nm; FM® 4–64 ex/em 485/>620 nm), and combined with differential interference contrast (DIC) images for ease of orientation. Exposure time and illumination parameters were adjusted individually for optimal visibility. For bud scar visualization, Z-stack image series of representative spots were recorded and processed via moderate iterative deconvolution. Lectin cytoadhesion was quantified by incubating the cells with the respective lectin solutions and FM® 4–64 as described above (4°C, 30 min), followed by extensive washing. Cells were then lysed by treatment with Triton X-100/SDS (1.0/1.0%) for 24 h under vivid agitation. The FITC fluorescence intensity in the lysis buffer was assessed in a microplate reader (TECAN, Austria) at ex/em 485/525 nm and normalized to the content of FM® 4–64 for direct comparison between the individual samples. Lectin solutions without cells were subjected to the same treatment and analyzed in order to exclude potential degradation of the fluorophore. Control experiments via fluorescence-activated cell sorting (FACS) were performed to verify similar uptake of the membrane stain in both strains.

#### Membrane permeabilization experiments

To gain information on the steric accessibility of cell wall-embedded chitin and other carbohydrates, cell permeability was enhanced by treatment with concentrated methanol at −20°C for 20 min, followed by lectin staining. The cells contained in 1.0 mL of precooled suspension (OD_600_ of 0.3) were harvested by centrifugation, resuspended in 100 μL PBS buffer and added dropwise to 1.0 mL of icecold methanol under vivid agitation. After 20 min, cells were pelleted again and excessive solvent was removed via repeated washing and centrifugation with fresh PBS buffer. After rehydration in 1.0 mL of 20 mM HEPES buffer, pH 7.4, cells were subjected to the same lectin staining protocol as described above and analyzed via fluorescence microscopy. Efficient membrane permeabilization was verified via successful counterstaining of the nuclear DNA with a normally non-membrane permeable DAPI dye.

### Production of the reporter enzyme horseradish peroxidase in shake flask experiments

Aliquots of approximately 2 μg of *SmiI* linearized plasmid pPpT4_S^18^, harboring a HRP gene containing nine potential N-glycosylation sites were transformed into either *Pp*MutS or *Pp*FWK3. The transformed HRP gene encodes for a new acidic HRP isoenzyme. A detailed description on the identification of new HRP isoenzymes will be given elsewhere (Näätsaari *et al.*, manuscript in preparation). The HRP gene was codon optimized for expression in *P. pastoris* based on a codon table described in^46^. Expression of the gene was regulated by the *AOX1* promoter. Efficient secretion of HRP to the supernatant was facilitated by fusion of the prepro signal sequence of the *S. cerevisiae* mating factor alpha to the N-terminus of the mature HRP. Transformations were performed according to^44^ with the following modification: Whereas an overnight culture of *Pp*MutS was diluted to an OD_600_ of 0.2 to grow to an OD_600_ of 0.8–1.0 in approximately 5 h prior to preparation of the cells for electroporation, an overnight culture of *Pp*FWK3 was diluted to a starting OD_600_ of 0.7 to account for its decreased growth rate. Transformants were grown on YPD Zeocin™ agar plates and randomly chosen for screening in micro scale cultivations in 96-deep well plates, similarly to^47^. The cells were cultivated in 250 μL iron-supplemented BMD1% (11 g·L^−1^ α-D(+)-glucose monohydrate, 13.4 g·L^−1^ YNB, 0.4 mg·L^−1^ D(+)-biotin, 278 mg·L^−1^ FeSO_4_ 7H_2_O, 0.1 M potassium phosphate buffer, pH 6.0) for approximately 60 h, then induced once with 250 μL BMM2 (1% (v/v) methanol, 13.4 g·L^−1^ YNB, 0.4 mg·L^−1^ D(+)-biotin, 0.1 M potassium phosphate buffer, pH 6.0) and three times with 50 μL BMM10 (5% (v/v) methanol, 13.4 g·L^−1^ YNB, 0.4 mg·L^−1^ D(+)-biotin, 0.1 M potassium phosphate buffer, pH 6.0) per well 12 h, 24 h and 36 h after the first addition of BMM2. Induction with the methanol containing media BMM2 and BMM10 induced the production of HRP which was under control of the *AOX1* promoter. The respective HRP production strains were designated *Pp*MutS^HRP^ and *Pp*FWK3^HRP^.

Small scale cultivations were performed in 0.5 L Ultra Yield Flasks (BioSilta, Finland) in 45 mL iron-supplemented BMD1%. After approximately 60 h, 5 mL BMM10 were added. Twelve hours and 36 h after the first induction pulse, 0.5 mL pure methanol were added. Twentyfour hours and 48 h after the first induction pulse, 0.25 mL pure methanol were added. HRP activity in the supernatant was determined by mixing 15 μL of culture supernatant with 140 μL of assay solution (1 mM ABTS, 0.8 mM H_2_O_2_, 50 mM NaOAc buffer, pH 4.5) and following the increase in absorbance at 405 nm in a Spectramax Plus 384 platereader (Molecular Devices, Germany) at room temperature for 3 min. Promising clones were streaked to single colonies and cultivated again in quadruplicates for rescreening. The copy number of the HRP gene in selected *Pp*MutS and *Pp*FWK3 transformant strains was determined via quantitiative real-time PCR according to a protocol of Abad *et al.*^48^ and as described previously in^26^.

### Bioreactor cultivations

Four different P. pastoris strains (Table 6) were characterized in terms of physiology, biomass growth and productivity by a novel, dynamic strategy of conducting methanol pulses during batch cultivations in the controlled environment of a bioreactor, which we have described recently^16^,^24^,^26^.

#### Culture media

Yeast nitrogen base medium (YNBM): 20 g·L^−1^ α-D(+)-glucose monohydrate, 3.4 g·L^−1^ YNB2, 10 g·L^−1^ (NH_4_)_2_SO_4_, 0.4 g·L^−1^ D(+)-biotin, 0.1 M potassium phosphate buffer, pH 6.0.

Trace element solution (PTM1): 6 g·L^−1^ CuSO_4_.5H_2_O, 0.08 g·L^−1^ NaI, 3 g·L^−1^ MnSO_4_·H_2_O, 0.2 g.L^−1^ Na_2_MoO_4_·2H_2_O, 0.02 g·L^−1^ H_3_BO_3_, 0.5 g·L^−1^ CoCl_2_, 20 g·L^−1^ ZnCl_2_, 65 g·L^−1^ FeSO_4_·7H_2_O, 0.2 g·L^−1^ D(+)-biotin, 5 mL·L^−1^ 95–98% H_2_SO_4_.

Basal salt medium (BSM): 44 g·L^−1^ α-D(+)-glucose monohydrate, 1.17 g·L^−1^ CaSO_4_·2H_2_O, 18.2 g·L^−1^ K_2_SO_4_, 14.9 g·L^−1^ MgSO_4_.7H_2_O, 4.13 g·L^−1^ KOH, 26.7 mL·L^−1^ 85% (v/v) o-phosphoric acid, 0.2 mL·L^−1^ Antifoam Struktol J650, 4.35 mL·L^−1^ PTM1, NH_4_OH as N-source (see experimental procedure).

Base: NH_4_OH, concentration was determined by titration with 0.25 M potassium hydrogen phthalate.

#### Preculture

Frozen stocks (−80°C) of either *Pp*MutS^HRP^ and *Pp*FWK3^HRP^ were precultivated in 100 mL of YNBM in 1 L shake flasks at 30°C and 230 rpm for max. 24 h. The preculture was transferred aseptically to the respective culture vessel. The inoculation volume was 10% of the final starting volume.

#### Batch cultivation

Batch cultivations were carried out in a 3 L working volume Labfors glass bioreactor (Infors, Switzerland). BSM was sterilized in the bioreactor and pH was adjusted to pH 5.0 by using concentrated ammonia solution after autoclaving. Sterile filtered PTM1 was transferred to the reactor aseptically. Dissolved oxygen (dO_2_) was measured with a sterilizable polarographic dissolved oxygen electrode (Mettler Toledo, Switzerland). The pH was measured with a sterilizable electrode (Mettler Toledo, Switzerland) and maintained constant with a step controller using 2.5 M ammonia solution. Base consumption was determined gravimetrically. Cultivation temperature was set to 30°C and agitation was fixed to 1495 rpm. The culture was aerated with 2.0 vvm dried air and offgas of the culture was measured by using an infrared cell for CO_2_ and a paramagnetic cell for O_2_ concentration (Servomax, Switzerland). Temperature, pH, dO_2_, agitation as well as CO_2_ and O_2_ in the offgas were measured online and logged in a process information management system (PIMS Lucullus; Biospectra, Switzerland).

After the complete consumption of the substrate glucose, which was indicated by an increase of dO_2_ and a drop in offgas activity, the first methanol pulse (adaptation pulse) of a final concentration of 0.5% (v/v) was conducted with methanol supplemented with PTM1 (12 mL PTM1 per 1 L of methanol). Subsequently, between five and seven pulses were performed with 1% or 2% (v/v) methanol for each strain. For each pulse, at least two samples were taken to determine the concentrations of the substrate methanol and product as well as dry cell weight (DCW) and OD_600_ to calculate the strain specific parameters. The induction period for *Pp*MutS^HRP^ and *Pp*FWK3^HRP^ was carried out in the presence of 1 mM of the heme precursor δ-aminolevulinic acid.

#### Analysis of growth- and expression-parameters

DCW was determined by centrifugation of 5 mL culture broth (4,000 × g, 10 min, 4°C), washing the pellet with 5 mL deionized water and subsequent drying at 105°C to a constant weight in an oven. OD_600_ of the culture broth was measured using a spectrophotometer (Genesys 20; Thermo Scientific, Austria). The activity of HRP was determined using a CuBiAn XC enzymatic robot (Innovatis, Germany). Cell free samples (10 μL) were added to 140 μL of 1 mM ABTS in 50 mM potassium phosphate buffer, pH 6.5. The reaction mixture was incubated at 37°C and was started by the addition of 20 μL of 0.075% H_2_O_2_. Changes of absorbance at 415 nm were measured for 80 s and rates were calculated. Calibration was done using commercially available horseradish peroxidase (Type VI-A, Sigma-Aldrich, Austria, P6782, Lot# 118K76734) as standard at six different concentrations (0.02; 0.05; 0.1; 0.25; 0.5 and 1.0 U·mL^−1^). Protein concentrations were determined at 595 nm using the Bradford Protein Assay Kit (Bio-Rad Laboratories GmbH, Austria) with bovine serum albumin as standard.

#### Substrate concentrations

Concentration of methanol was determined in cell free samples by HPLC (Agilent Technologies, USA) equipped with a Supelcoguard column, a Supelcogel C-610H ion-exchange column (Sigma-Aldrich, Austria) and a refractive index detector (Agilent Technologies, USA). The mobile phase was 0.1% H_3_PO_4_ with a constant flow rate of 0.5 mL·min^−1^ and the system was run isocratically. Calibration was done by measuring standard points in the range of 0.1 to 10 g·L^−1^ methanol.

#### Data analysis

Strain characteristic parameters were determined at a carbon dioxide evolution rate (CER) above 2.5 mmol·L^−1^·h^−1^ during each methanol pulse. Measurements of biomass, product and substrate concentration were executed in duplicates. Along the observed standard deviation for the single measurement, the error was propagated to the specific rates q_s_ and q_p_ as well as to the yield coefficients. The error of determination of the specific rates and the yields was therefore set to 10% and 5%, respectively^16^,^24^.

### Enzyme purification

#### Size exclusion chromatography

The supernatants from *Pp*MutS^HRP^ and *Pp*FWK3^HRP^ produced in small scale cultures in 0.5 L Ultra Yield Flasks were concentrated to approximately 500 μL each using Vivaspin 20 tubes (Sartorius Stedim Biotech, Germany) with 10 kDa MWCO and recovered from the tubes resulting in a volume of max. 1500 μL, prior to size exclusion chromatography (SEC) on a HiLoad™ 16/60 Superdex 200 prep grade column (GE Healthcare Europe, Austria). SEC was performed at a flow rate of approximately 9 cm·h^−1^, fractions of 1.2 mL were collected and assayed for HRP activity using ABTS as substrate.

#### 2-step purification protocol

To purify the secreted HRP produced in bioreactor cultivations, the fermentation broths were harvested and centrifuged (4,000 × g, 20 min) and the cell free supernatants were subjected to diafiltration with buffer (500 mM NaCl, 20 mM NaOAc, pH 6.0) for a subsequent purification step via a mixed mode resin (hydrophobic charge induction chromatography, HCIC) followed by a size exclusion step (SEC). We have recently described this 2-step flowthrough based strategy for the HRP isoenzyme C1A^27^. The catalytic activity and the protein content in all fractions were determined, active fractions were pooled and concentrated via ultrafiltration to approximately 3 mg·mL^−1^ for HRP produced in *Pp*Muts^HRP^ and 0.3 mg·mL^−1^ for HRP produced in *Pp*FWK3^HRP^ for subsequent enzyme characterization.

### Enzyme characterization

The two HRP preparations produced in either *Pp*Muts^HRP^ or in *Pp*FWK3^HRP^ were characterized to determine differences between the hypermannosylated HRP from *Pp*MutS^HRP^ and its glycovariant produced in *Pp*FWK3^HRP^.

#### Liquid chromatography-mass spectrometry (LC-MS) analysis

Protein N-glycosylation was analyzed by releasing the N-glycans with peptide:N-glycosidase F (Roche, Mannheim). The released N-glycans were desalted and analyzed using a porous graphitic carbon capillary column (ThermoScientific) coupled to a mass spectrometer (Maxis 4 G, Bruker, Bremen). Deviating from previous work^49^, glycans were not reduced and a steep gradient was applied leading to the elution of all glycans within approximately 2 min.

#### Kinetic constants with ABTS

Protein concentrations of the HRP preparations were determined at 595 nm using the Bradford Protein Assay Kit (Bio-Rad Laboratories GmbH, Austria) with bovine serum albumin as standard. The kinetic constants for ABTS were determined for both HRP glycovariants. The reaction was started by adding 10 μL enzyme solution (3 mg·mL^−1^ HRP from *Pp*MutS^HRP^ and 0.3 mg·mL^−1^ HRP from *Pp*FWK3^HRP^) to 990 μL reaction buffer containing ABTS in varying concentrations (0.01–10 mM), 1 mM H_2_O_2_ and 50 mM potassium phosphate, pH 6.5. The change in absorbance at 420 nm was recorded in a spectrophotometer UV-1601 (Shimadzu, Japan) at 30°C controlled with a temperature controller (CPS controller 240 A; Shimadzu, Japan). Absorption curves were recorded with a software program (UVPC Optional Kinetics; Shimadzu, Japan). Measurements were performed in triplicates.

#### Thermal stability

Both enzyme solutions were incubated at 60°C for 1 h. At different time points, aliquots were withdrawn, the solutions were immediately cooled and centrifuged (20,000 × g, 15 min) to pellet precipitated proteins and the remaining catalytic activity in the supernatants was measured^28^.

## Author Contributions

F.W.K. and O.S. conceived of and planned the study. F.W.K., C.G., L.N., M.W., R.P. and O.S. conducted the different experiments. C.H., A.G. and F.A. supervised parts of the research. F.W.K. and O.S. wrote the paper.

## Supplementary Material

Supplementary Information Supplementary Info

## Figures and Tables

**Figure 1:**
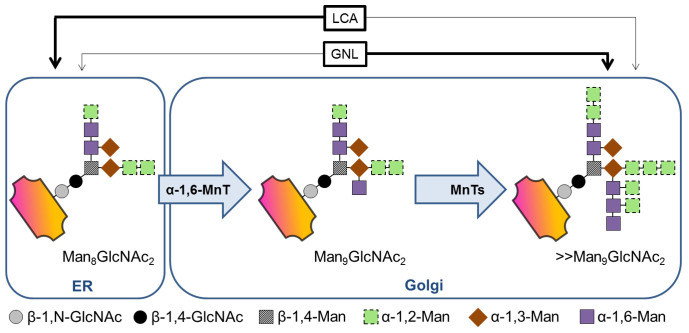
Och1p in N-glycan biosynthesis. In the Golgi, the α-1,6-mannosyltransferase activity (α-1,6-MnT) of Och1p extends the N-linked Man_8_GlcNAc_2_ core glycan, which is then heterogeneously hyperglycosylated by several additional (phospho-) mannosyltransferases (MnTs). *Galanthus nivalis* lectin (GNL) and *Lens culinaris* lectin (LCA) bind to the different glycan structures either with high (thick arrow) or low (thin arrow) specificity.

**Table 1:** Humanization of N-glycans in *P. pastoris.* Selected studies focusing on the humanization of the N-glycans on *P. pastoris* derived glycoproteins. Bmt, β-mannosyltransferase; Mns, mannosidase; GnT, β-N-acetylglucosaminyltransferase; UDP-GlcNAc, uridine diphosphate-N-acetylglucosamine; *OCH1*, outer chain elongation gene 1

content	references
introduction of α-1,2-Mns, GnTI and an UDP-GlcNAc transporter via a combinatorial genetic library approach in a Δ*och1*::*URA3* strain	13,50
introduction of an UDP-GlcNAc transporter, α-1,2-MnsIA, MnsII, GntI, GntII in a Δ*och1*::*URA3* strain	51
introduction of α-1,2-Mns and GnTI, *OCH1* inactivation via a knockin plasmid	10
GlycoSwitch plasmids for *OCH1* inactivation and introduction of glycosidase and glycosyltransferase activities to produce complex terminally galactosylated glycoproteins	52
introduction of sialic acid biosynthesis pathway and corresponding transporter and transferase activities to produce complex terminally sialylated glycoproteins	53
elimination of α-Mns resistant glycan structures by inactivation of the activities of Bmt1p, Bmt2p and Bmt3p	54

**Table 2:** Strain specific parameters of the different *P. pastoris* strains. Parameters are defined in the footnote^x^

	*Pp*MutS	*Pp*MutS^HRP^	*Pp*FWK3	*Pp*FWK3^HRP^
**max. μ_Gly_ (h^−1^)**	0.30	0.31	0.20	0.20
**q_Gly_ (mmol·g^−1^·h^−1^)**	2.90	3.10	1.90	1.90
**Y_X/Gly_ (Cmol·Cmol^−1^)**	0.63	0.41	0.61	0.54
**Y_CO2/Gly_ (Cmol·Cmol^−1^)**	0.33	0.64	0.37	0.44
**Δtime_adapt_ (h)**	15.7	19.9	6.60	8.90
**q_MeOH_ (mmol·g^−1^·h^−1^)**	0.62	0.70	0.52	0.43
**max. q_MeOH-_(mmol·g^−1^·h^−1^)**	0.67	0.78	0.69	0.53
**Y_X/MeOH_ (Cmol·Cmol^−1^)**	0.39	0.07	0.05	0.04
**Y_CO2/MeOH_ (Cmol·Cmol^−1^)**	0.57	1.02	constantly decreasing	constantly decreasing
**C-balance**	0.97	1.04	constantly decreasing	constantly decreasing
**q_p_ (U·g^−1^·h^−1^)**	-	0.77	-	0.50
**vol. productivity (U·L^−1^·h^−1^)**	-	2.60	-	1.80
**efficiency factor (η) (U·mmol^−1^** 26**)**	-	1.10	-	1.20

^X^max. μ_Gly_, maximum specific growth rate on glycerol; q_Gly_, specific uptake rate of glycerol during the batch; Y_X/Gly_, biomass yield on glycerol; Y_CO2/Gly_, CO_2_ yield on glycerol; Δtime_adapt_, time from first addition of methanol to a maximum in offgas activity; q_MeOH_, average specific uptake rate of methanol during consecutive methanol pulses; max. q_MeOH_, maximum specific uptake rate of methanol during consecutive methanol pulses; Y_X/MeOH_ biomass yield on methanol; Y_CO2/MeOH_, CO_2_ yield on methanol; C-balance, sum of biomass and CO_2_ yields; q_p_, specific productivity of HRP; vol. productivity, volumetric productivity of HRP; efficiency factor; efficiency of the conversion of substrate methanol into product HRP.

**Table 3:** Relative peak areas of identified glycan structures cleaved off from HRP recombinantly produced in either *Pp*MutS^HRP^ or in *Pp*FWK3^HRP^

	*Pp*MutS^HRP^	*Pp*FWK3^HRP^
glycan structure	relative peak area [%]	
Man7	0.0	7.9
Man8	0.0	57.3
Man9	15.7	16.8
Man10	31.4	6.1
Man10-P	5.6	0.0
Man11	18.7	6.8
Man11-P	6.7	0.0
Man12	6.7	3.2
Man12-P	4.4	0.0
Man13	3.7	1.8
Man13-P	1.8	0.0
Man14	2.1	0.0
Man14-P	1.1	0.0
Man15	1.2	0.0
Man16	0.5	0.0
Man17	0.4	0.0
total	100.0	100.0

**Table 4:** Enzymatic characterization of homogeneously glycosylated HRP. Kinetic constants of HRP produced in either *Pp*MutS^HRP^ or *Pp*FWK3^HRP^ with H_2_O_2_ as electron donor at saturating concentration and ABTS as electron acceptor in varying concentrations were determined at 420 nm and 30°C

production strain	K_M_ABTS_ [mM]	V_max_ [μmol·s^−1^·μg^−1^]
*Pp*MutS^HRP^	2.40	3.07
*Pp*FWK3^HRP^	2.03	2.46

**Table 5:** Oligonucleotide primer list. Primers used for the amplification of upstream and downstream sequences of the *OCH1* locus from genomic DNA, for amplification of the whole *OCH1* locus for Sanger sequencing, for amplification of fragments to assemble pPpT4_BamHI_OCH1rescue and to amplify the *OCH1* ORF

primer name	sequence (5′ - 3′)
OCH1-5int-fw1	GAACTGTGTAACCTTTTAAATGACGGGATCTAAATACGTCATG
OCH1-5int-rv1	CTATTCTCTAGAAAGTATAGGAACTTCGGCTGATGATATTTGCTACGAACACTG
OCH1-3int-fw1b	GTTCCTATACTTTCTAGAGAATAGGAACTTCGCGAGATTAGAGAATGAATACCTTCTTCTAAGCGATCG
OCH1-3int-rv1b	GAAGTATTAGGAGCTGAAGAAGCAGAGGCAGAG
OCH1check-fw1	CACACATATAAAGGCAATCTACG
OCH1check-rv2	CAATAACTTCTGCAATAGACTGC
OCH1rescue-fw1	TTCATAGGCTTGGGGTAATAG
OCH1rescue-rv1	CTTGAGCGGCCGCTTAGTCCTTCCAACTTCCTTC
OCH1rescue_T4fw	GCATACATTTGAAGGAAGTTGGAAGGACTAAGCGGCCGCTCAAGAGGAT
OCH1rescue_T4rv	CTATTTCTCTGTCATCTATCTATTACCCCAAGCCTATGAAGGATCTGGGTACCGCAGG
OCH1-ORF-fw	ATGGCGAAGGCAGATGGC
OCH1-ORF-rv	TTAGTCCTTCCAACTTCCTTCAAATG

**Table 6:** *P. pastoris* strains used for biotechnological characterisation during bioreactor cultivations

strain	name
*P. pastoris* Mut^S^	*Pp*MutS
*P. pastoris* Mut^S^ HRP	*Pp*MutS^HRP^
*P. pastoris* Mut^S^ *och1*	*Pp*FWK3
*P. pastoris* Mut^S^ *och1* HRP	*Pp*FWK3^HRP^
