# Supplementary material for: Knockout of an endogenous mannosyltransferase increases the homogeneity of glycoproteins produced in Pichia pastoris
Source: Sci Rep. 2013 Nov 20;3:3279. doi: 10.1038/srep03279 (PMC3834888; doi:10.1038/srep03279)
Supplement: Supplementary Information — Supplementary Info [file srep03279-s1.pdf]

# Knockout of an endogenous mannosyltransferase increases the homogeneity of glycoproteins produced in *Pichia pastoris*

Florian W. Krainer[1], Christoph Gmeiner[2], Lukas Neutsch[3], Markus Windwarder[4], Robert Pletzenauer[2], Friedrich Altmann[4], Anton Glieder[5] and Oliver Spadiut[2*]

## Supplementary Figures

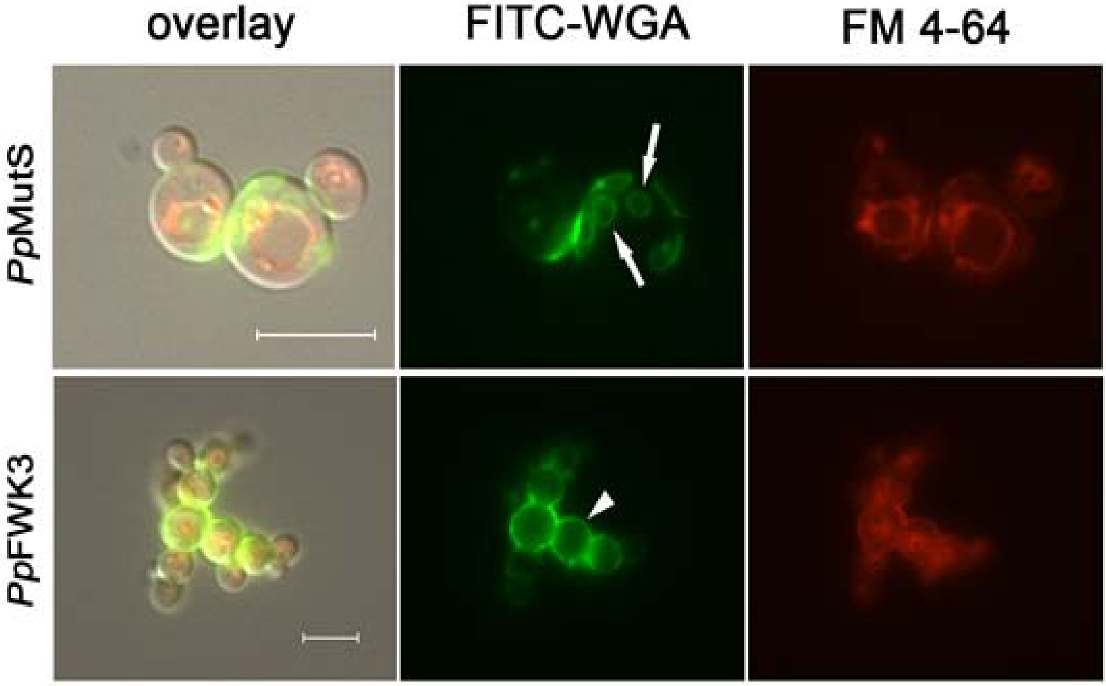


**Supplementary Figure 1.** Differential chitin deposition in response to *OCH1* knockout. Surface accessible chitin was visualized via FITC-WGA staining (250 pmol·mL$^{-1}$) of live yeast cells, corresponding to either the *Pp*MutS strain (upper panel) or the *Pp*FWK3 strain (lower panel). Vacuoles were counterstained with the lipophilic membrane dye FM® 4-64. Fluorescence micrographs show the individual channels (middle, right) and an overlay DIC

image (left) for ease of orientation. Note the bud scar selective localization of chitin in the wildtype strain (arrows), in contrast to the non-specific deposition pattern in the entire cell wall after knockout of Och1p (arrowhead). Scale bars represent 5 µm.

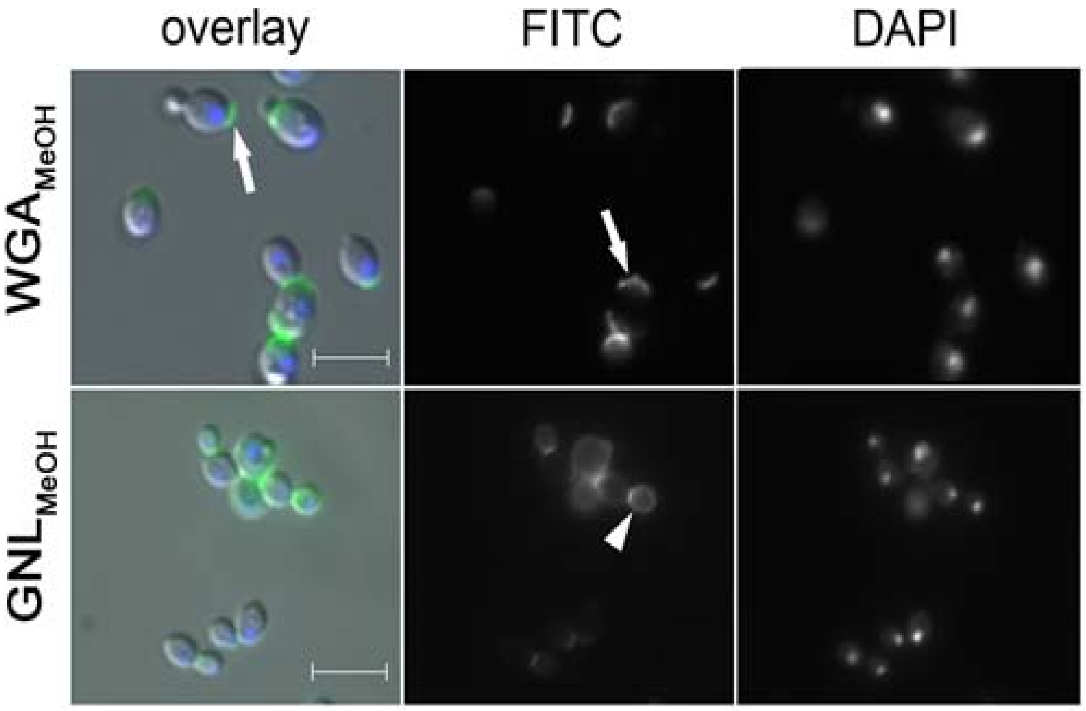


**Supplementary Figure 2.** Staining pattern of FITC-labeled chitin- (WGA) and mannosyl-specific (GNL) lectins in *Pp*MutS after cell permeabilization with icecold methanol. A non-membrane penetrating DNA stain was included as a control for successful cell wall permeabilization (DAPI). Images show the individual fluorescence channels (FITC, DAPI) and an overlay with DIC micrographs. Exposure times were individually adjusted for optimal visualization of the regional distribution. WGA binding was still confined to the bud scar regions (arrows), while GNL was distributed throughout the entire cell wall (arrowhead). This corresponded to the staining pattern obtained without prior permeabilization of the cell wall. Scale bars represent 10 µm.

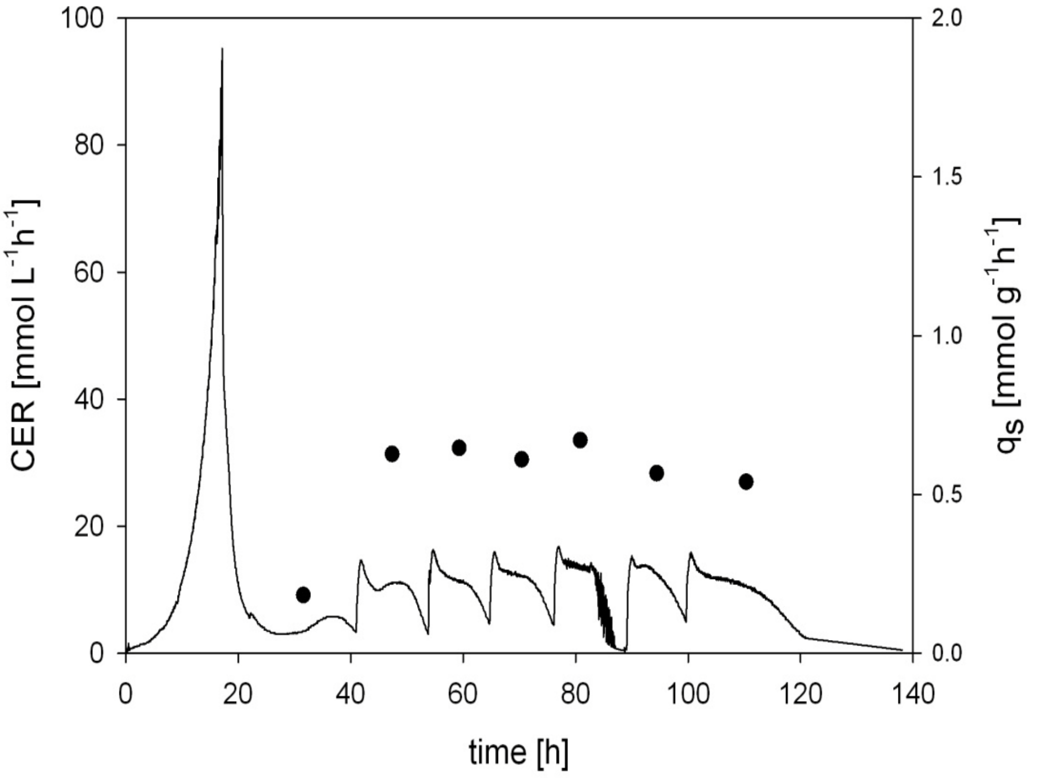


**Supplementary Figure 3.** Batch cultivation of strain *Pp*MutS with a methanol adaptation pulse of 0.5 %, subsequent 1 % pulses and a last 2 % pulse (v/v). Solid line, carbon dioxide evolution rate (CER); black dot, specific substrate uptake rate ($q_s$).

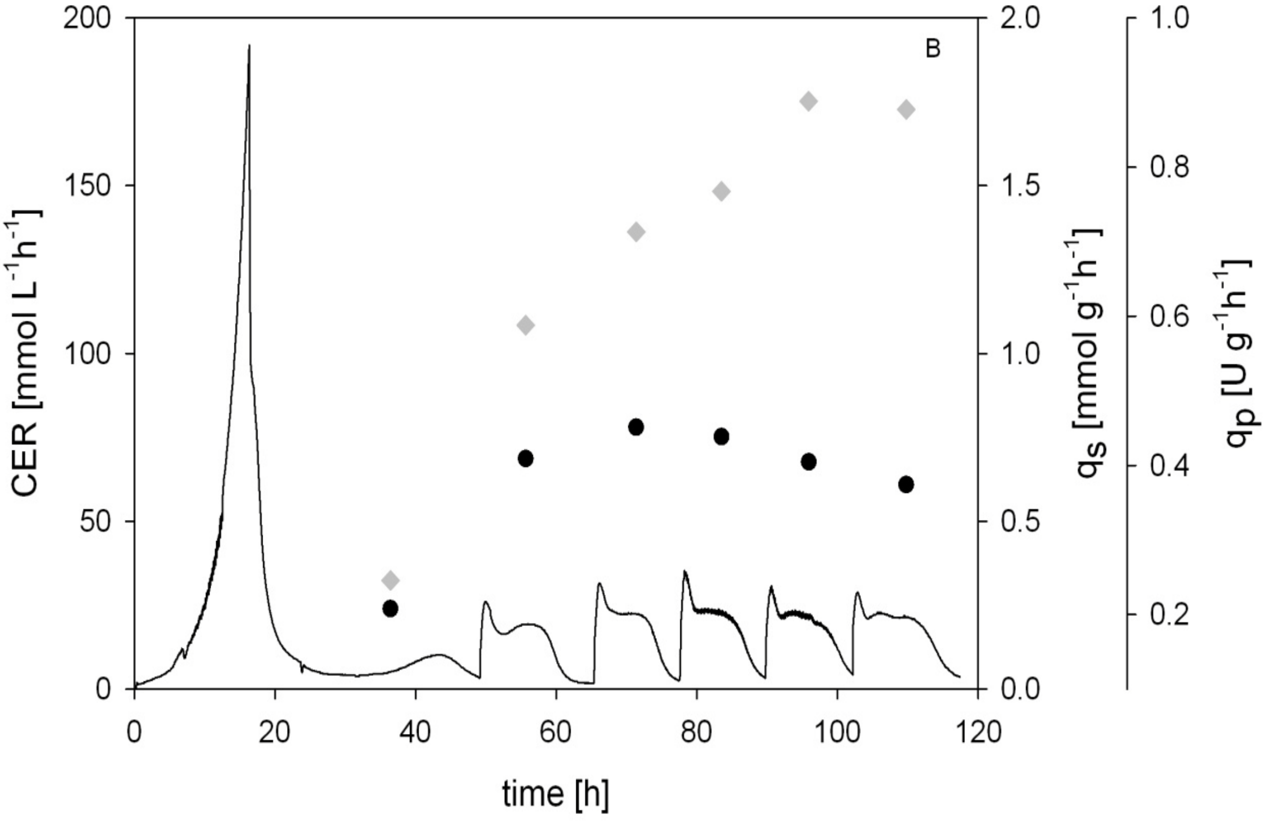


**Supplementary Figure 4.** Batch cultivation of strain *Pp*MutS$^{HRP}$ with a methanol adaptation pulse of 0.5 % and subsequent 1 % pulses (v/v). Solid line, carbon dioxide evolution rate (CER); black dot, specific substrate uptake rate ($q_s$), grey diamond, specific productivity ($q_p$).

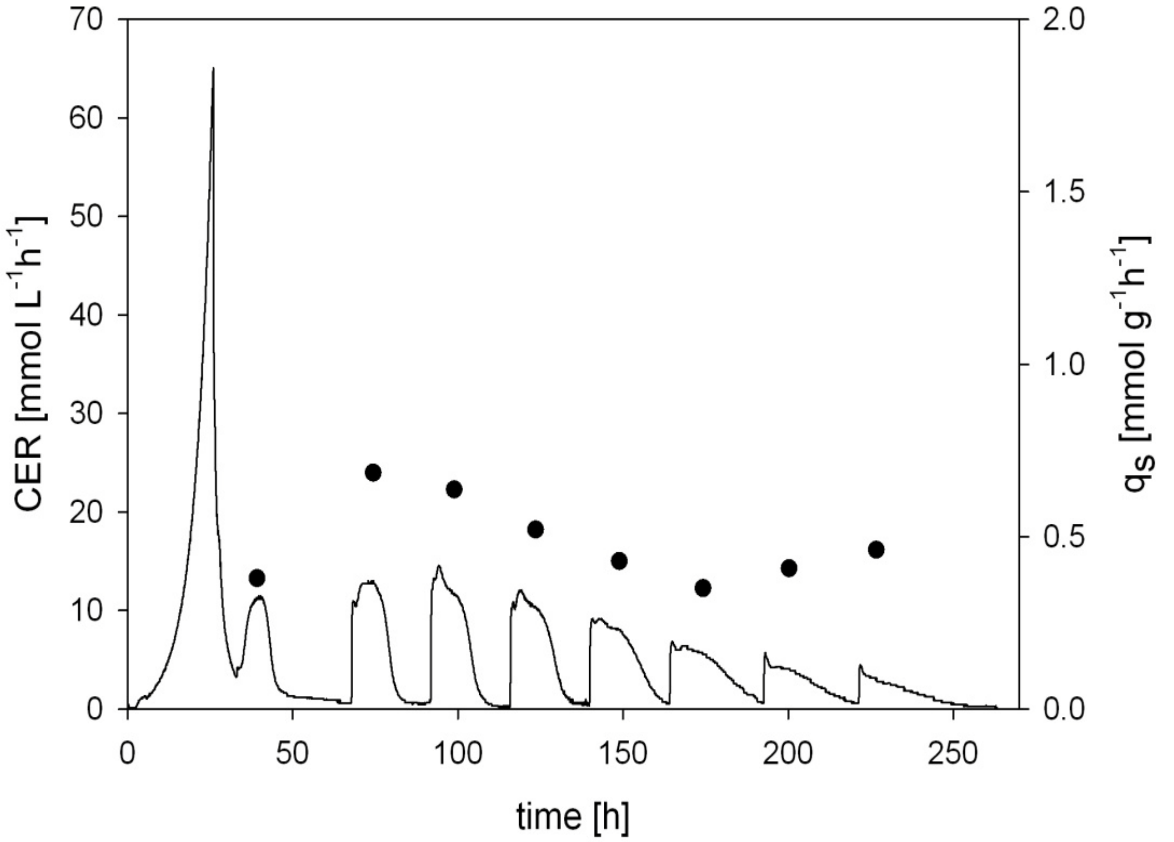


**Supplementary Figure 5.** Batch cultivation of strain *Pp*FWK3 with a methanol adaptation pulse of 0.5 % and subsequent 1 % pulses (v/v). Solid line, carbon dioxide evolution rate (CER); black dot, specific substrate uptake rate ($q_S$).

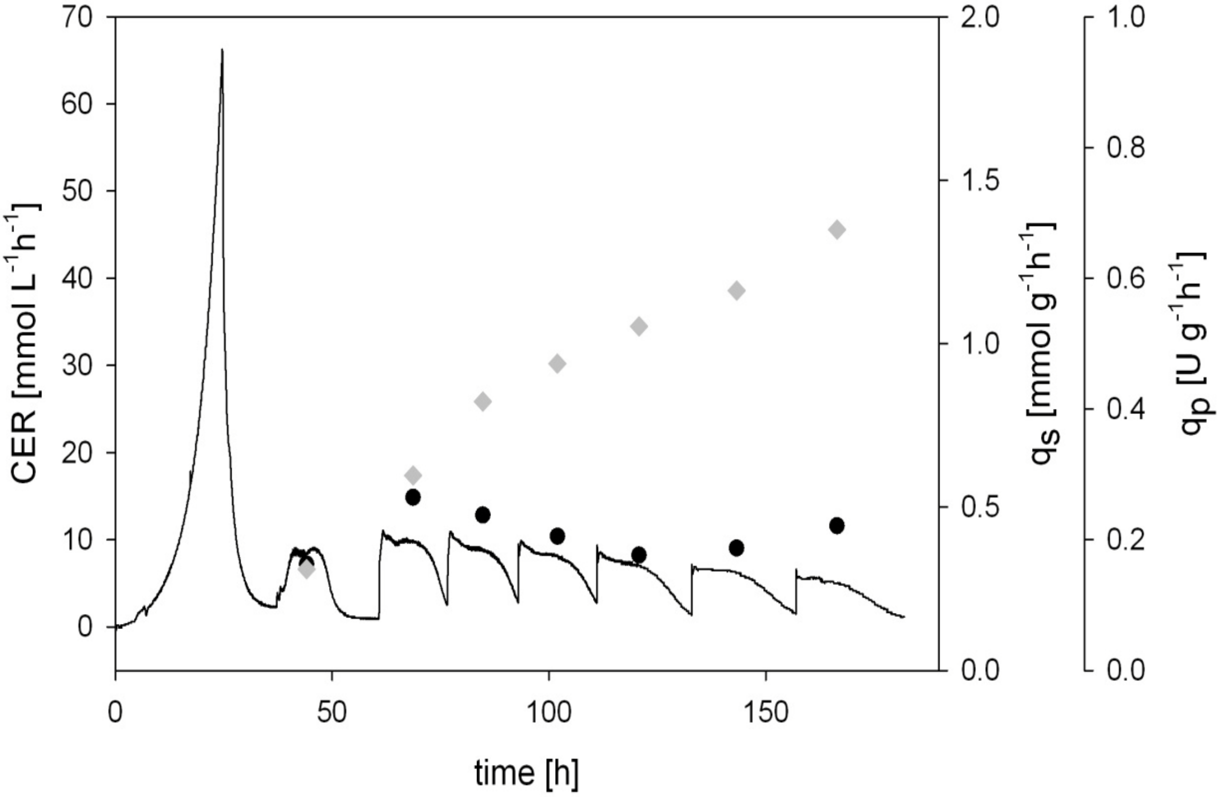


**Supplementary Figure 6.** Batch cultivation of strain *Pp*FWK3$^{HRP}$ with a methanol adaptation pulse of 0.5 % and subsequent 1 % pulses (v/v). Solid line, carbon dioxide evolution rate (CER); black dot, specific substrate uptake rate ($q_s$), grey diamond, specific productivity ($q_p$).

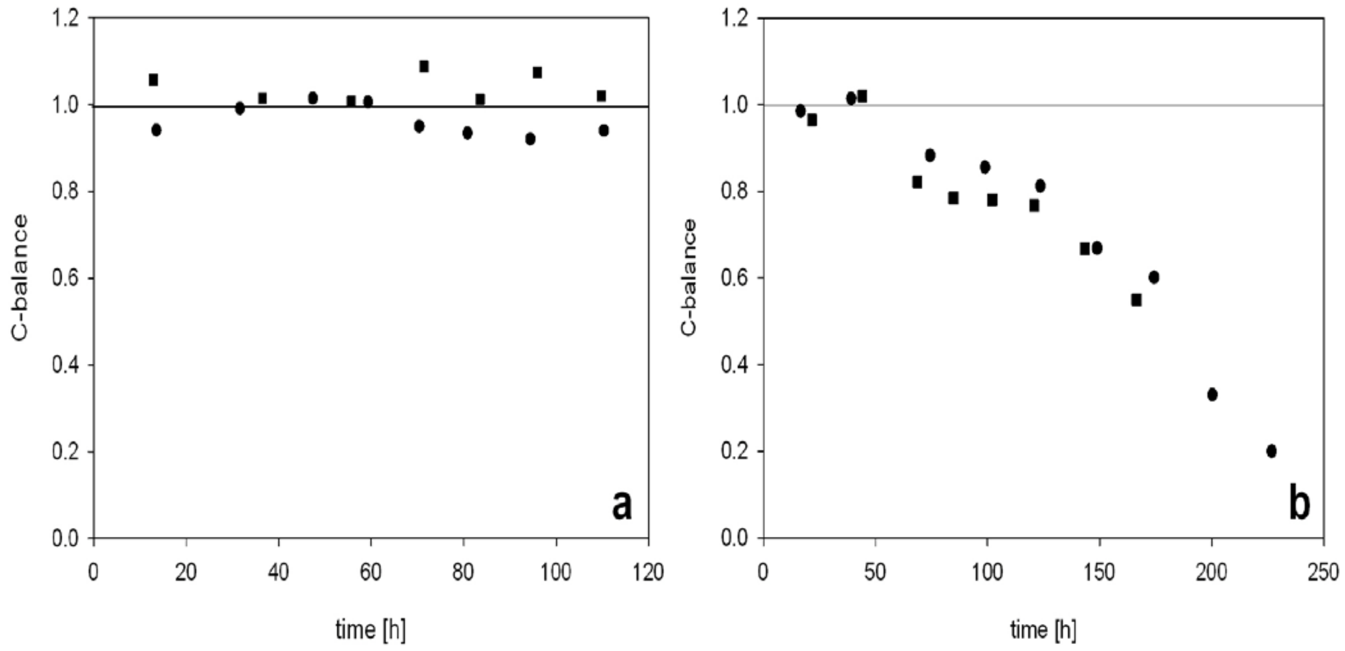


**Supplementary Figure 7.** C-balances of the different *P. pastoris* strains during the consecutive methanol pulses. A, *Pp*MutS strains; black dot, *Pp*MutS; black square, *Pp*MutS$^{HRP}$; B, *Pp*FWK3 strains; black dot, *Pp*FWK3; black square, *Pp*FWK3$^{HRP}$.

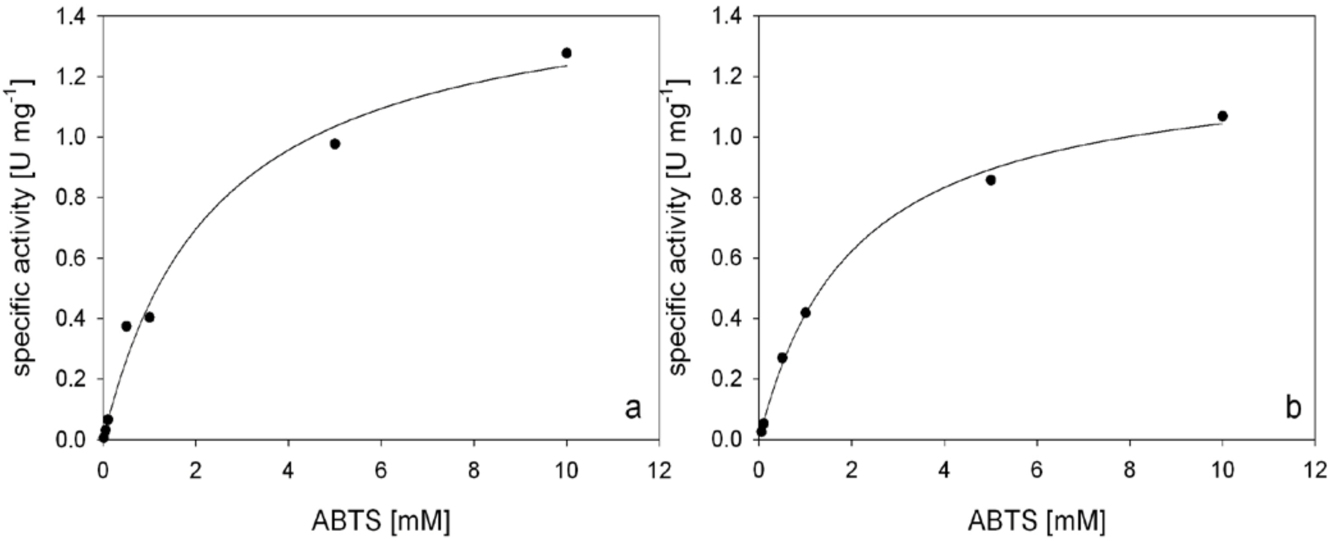


**Supplementary Figure 8.** Michaelis-Menten curves of HRP with $H_2O_2$ as electron donor in a saturating concentration of 1 mM and ABTS as electron acceptor in varying concentrations between 0.1 and 10.0 mM. The reaction was started by adding 10 μL enzyme solution to 990 μL reaction buffer containing ABTS, $H_2O_2$ and 50 mM potassium phosphate, pH 6.5. The change in absorbance at 420 nm was recorded in a spectrophotometer UV-1601 at 30 °C. Absorption curves were recorded with a software program (UVPC Optional Kinetics; Shimadzu, Japan). Measurements were performed in triplicates.

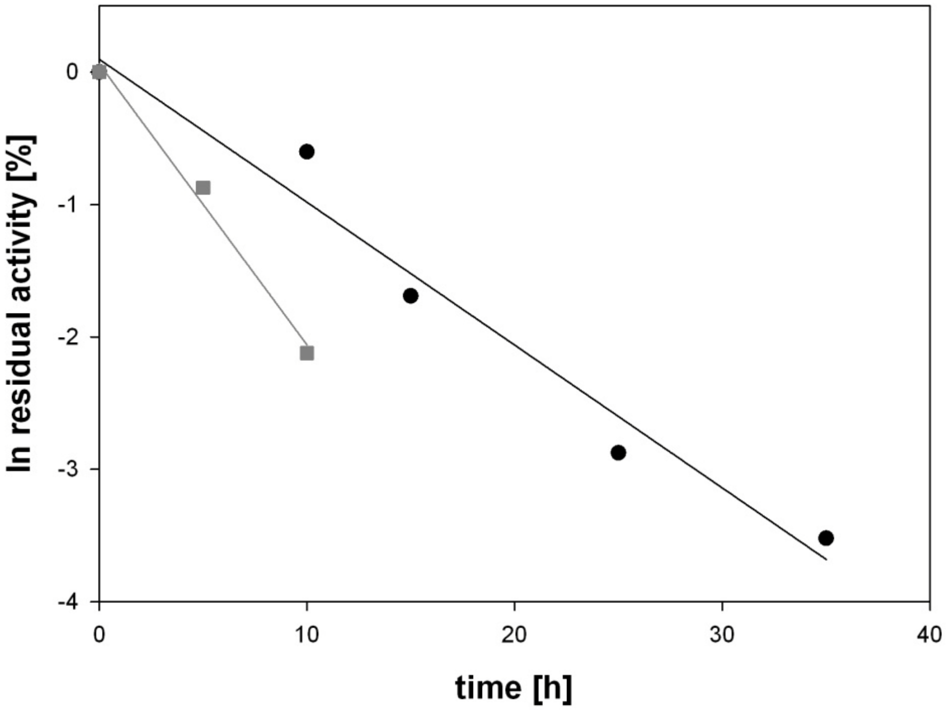


**Supplementary Figure 9.** Thermal stability of HRP glycovariants at 60 °C. Black dots and line, HRP produced in strain *Pp*MutS$^{HRP}$; dark grey dots and line, HRP produced in *Pp*FWK3$^{HRP}$.
